# Convergent Total Synthesis of PM742 and SAR-Guided Development of the Clinical Candidate PM534

**DOI:** 10.3390/md24050167

**Published:** 2026-05-07

**Authors:** María Jesús Martín, Juan Hernando, Raquel Rodríguez-Acebes, Asier Gómez-SanJuan, Andrés Francesch, Simon Munt, Carmen Cuevas

**Affiliations:** Research and Development, PharmaMar S.A., Avda. de los Reyes 1, Pol. In. La Mina, Colmenar Viejo, 28770 Madrid, Spain; mjmartin@pharmamar.com (M.J.M.); jhernando@pharmamar.com (J.H.); rrodriguez@pharmamar.com (R.R.-A.); agsanjuan@pharmamar.com (A.G.-S.); afrancesch@pharmamar.com (A.F.); smunt@pharmamar.com (S.M.)

**Keywords:** antitumor compounds, convergent synthesis, structure-activity relationship (SAR), in vitro cytotoxicity, total synthesis, phase I clinical trial

## Abstract

We report the first total synthesis of PM742 using N-Boc-D-Norvaline and 2-Methyl-L-Cysteine hydrochloride as starting materials in a convergent sequence of eight chemical steps. This strategy provides efficient access to PM742 and enables the preparation of structurally related analogs. PM742 exhibited in vitro cytotoxic activity against a panel of human tumor cell lines, which prompted preliminary structure-activity relationship (SAR) studies. These efforts led to the preparation of PM534, a related analog with improved activity that has now entered Phase I clinical trials in humans.

## 1. Introduction

**Figure 1:**
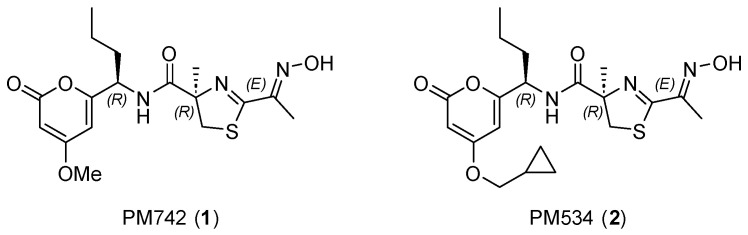
Chemical Structures of PM742 (**1**) and PM534 (**2**).

In our ongoing efforts to identify anticancer compounds from marine sources, we investigated the chemical composition of a *Discodermia du Bocage* specimen collected in the Pacific Ocean. This study led to the discovery of a new chemical entity, PM742 (**1**) (Figure 1), together with several analogs of the known aurantosides [1]. The planar structure of PM742 (**1**) consists of an α-pyrone linked through an amide bond to a 4-methyl-4,5-dihydrothiazole-derived E-oxime. Its structure was unequivocally confirmed by total synthesis, starting from N-Boc-D-Norvaline and 2-methyl-L-cysteine hydrochloride in a convergent sequence of eight chemical steps.

The biological activity of PM742 (**1**) was evaluated against a panel of human cancer cell lines, revealing strong in vitro cytotoxicity. These results prompted an extensive structure-activity relationship (SAR) study that ultimately led to the identification of PM534 (**2**) (Figure 1), a synthetic analog of PM742 (**1**) [2] with potent antitumor activity in both lung epithelial cells and non-small cell lung cancer (NSCLC) cells. Notably, PM534 (**2**) is able to overcome typical resistance mechanisms against microtubule-targeting agents (MTAs), including detox pump overexpression and the presence of the βIII-tubulin isotype [3].

PM534 (**2**) demonstrates strong antitumor activity across multiple tumor types, including those characterized by the presence of well-established multidrug resistance (primary or acquired) mechanisms. The antitumor activity stems from PM534’s ability to simultaneously target two critical cellular processes, namely tumor cell division and tumor vasculature, resulting in apoptosis and necrosis [4]. The development of an efficient synthetic route resolved the supply challenge and, together with comprehensive pharmaceutical and preclinical studies, enabled PM534 (**2**) to advance into clinical evaluation as a promising new anticancer drug. A first-in-human clinical trial (ClinicalTrials.gov Identifier: NCT05835609), initiated in December 2022, aims to determine the recommended dose and to evaluate the safety and preliminary efficacy profile of PM534 (**2**) in patients [5].

## 2. Results and Discussions

### 2.1. Total Synthesis of PM742 (***1***)

**Figure 2:**
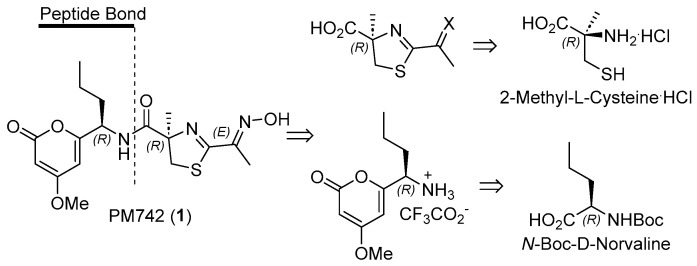
Retrosynthetic analysis of PM742 (**1**).

To overcome the supply limitations associated with the natural source and to enable further pharmaceutical development and in vivo preclinical studies, we have accomplished the first total synthesis of PM742 (**1**). Our strategy relied on a retrosynthetic analysis featuring a key disconnection that divides PM742 into two building blocks joined through a peptide bond, thus enabling an efficient convergent synthesis (Figure 2). One fragment, the methyl-thiazole derivative, was prepared from L-cysteine, while the second fragment, the pyrone moiety, was obtained using D-Norvaline as the starting material.

**Scheme 1:**
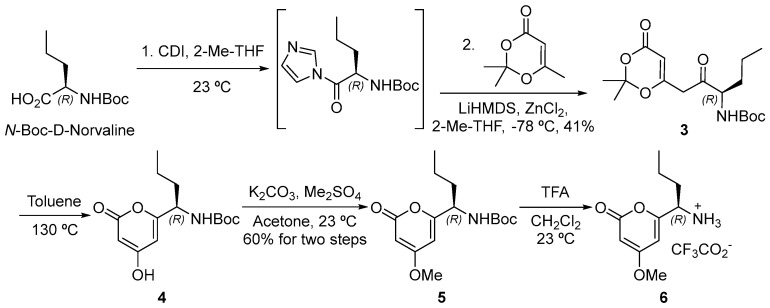
Synthesis of intermediate **6**.

Regarding the pyrone moiety, the main challenge was constructing the pyrone ring without causing epimerization of the starting amino acid D-Norvaline. Among the various synthetic approaches evaluated for the preparation of 2-pyrones, dimethyl-1,3-dioxinone (Scheme 1) provided the best results [6]. Thermal ring opening of 1,3-dioxinone **3** facilitated the subsequent cyclization to **4**, and the subsequent hydroxy group alkylation step to **5** was followed by *N*-Boc deprotection under acidic conditions to furnish the key pyrone fragment **6**.

Among the various synthetic routes described in the literature for preparing thiazolines, we selected the approach based on the reaction between 2-methyl-L-cysteine hydrochloride and a nitrile reagent. Specifically, using 2,2-diethoxypropionitrile as the ketone precursor, its reaction with 2-methyl-L-cysteine hydrochloride afforded the desired key methyl-thiazoline intermediate **7**. This amino acid is commercially available, and L-cysteine is an ideal precursor for the synthesis of α-methyl-L-cysteine through α-carbon methylation. To control the stereochemistry at this center, L-cysteine methylation is carried out only after the introduction of a temporary second chiral center following a known four-step sequence [7]. A large-scale preparation of 2-methyl-L-cysteine hydrochloride has also been reported as part of the multigram synthesis of Largazole [8].

**Scheme 2:**
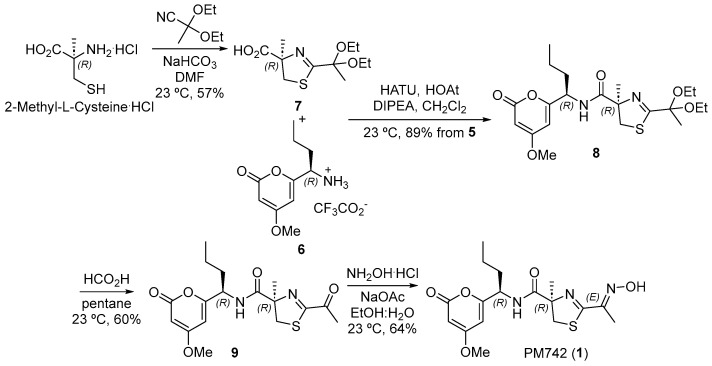
Final steps of PM742 (**1**).

The final three steps begin with the coupling of building blocks **6** and **7** (Scheme 2). Treatment of the resulting compound **8** with formic acid cleanly generated the desired ketone **9**, which subsequently reacted with hydroxylamine to yield predominantly the *E*-isomeric oxime; the *Z* isomer was formed as a minor byproduct during oxime formation, with an approximate *E*:*Z* ratio of 90:10. Overall, this efficient convergent route enables the straightforward preparation of PM742 (**1**), providing reliable access to the material required for the subsequent stages of drug development.

PM742 (**1**) attracted our interest as a novel chemical entity due to its potent in vitro antitumor activity and preliminary evidence suggesting a unique tubulin depolymerization mechanism. These attributes prompted the initiation of a drug development program. Leveraging our highly convergent synthetic route, we embarked on an extensive structure-activity relationship (SAR) study to identify the positions amenable to modification for enhanced activity and improved solubility. Consequently, a new series of analogs was designed, synthesized, and evaluated against human cancer cell lines for lead optimization [2].

### 2.2. Total Synthesis of PM534 (***2***)

**Scheme 3:**
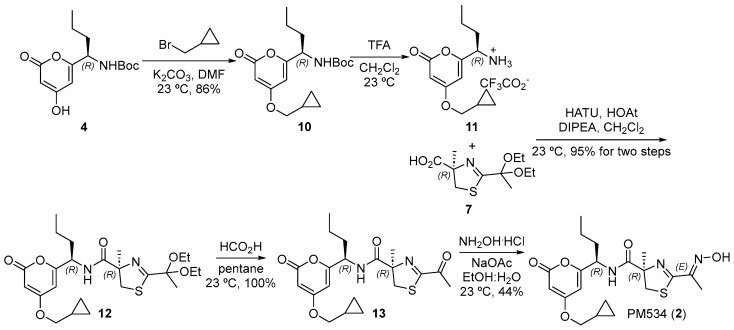
Synthesis of PM534 (**2**).

More than 100 analogs were synthesized for in vitro evaluation, providing extensive SAR data that guided the identification of more potent and selective compounds. Our convergent synthetic strategy enabled independent modification of the two building blocks, allowing multiple combinations to be explored. This approach revealed that certain molecular features, such as the norvaline residue and the methyl-thiazole moiety, are essential for activity, whereas others, including the oxime and hydroxy groups, can be modified to generate analogs with comparable or improved potency relative to the natural product. Based on both in vitro and in vivo antitumor activity, PM534 (**2**), bearing a cyclo-propyl group in place of the methyl group on the phenol, emerged as the lead candidate. The total synthesis of PM534 (**2**), starting from intermediates **7** and **10**, is outlined in Scheme 3.

The total synthesis of PM534 was accomplished through a multistep sequence starting from intermediates **7** and **10**. Key transformations included the preparation of the cyclo-propyl-substituted pyrone derivative **10** by alkylation with (bromomethyl)cyclopropane (86% yield), *N*-Boc deprotection of **10** with trifluoroacetic acid to give ammonium tri-fluoroacetate **11**, and peptide-bond formation with the methyl-thiazole fragment **7** to afford intermediate **12** (95% overall yield for the two steps). Subsequent treatment with formic acid provided quantitative conversion to ketone **13**, which was finally transformed into the *E*-oxime PM534 using hydroxylamine hydrochloride and sodium acetate; the *Z* isomer was formed as a minor byproduct during oxime formation, with an approximate *E*:*Z* ratio of 83:17. This convergent strategy supports scalable production for preclinical and clinical studies. The combination of synthetic accessibility, potent cytotoxicity, and favorable SAR underscores PM534 (**2**) as a promising candidate for anticancer drug development.

### 2.3. Biological Evaluation

The in vitro cytotoxic activity of PM742 (**1**), (*Z*)-PM742, PM534 (**2**), and (*Z*)-PM534 was evaluated against a panel of four human cancer cell lines, including A549 (lung), HT29 (colon), MDA-MB-231 (breast), and PSN (pancreas) (Table 1). PM742 (**1**) exhibited GI_50_ values of 11.5 nM (A549), 14.4 nM (HT29), 34.1 nM (MDA-MB-231), and 28.8 nM (PSN). Its *Z* isomer, (*Z*)-PM742, showed reduced potency with GI_50_ values of 144 nM, 152 nM, 204 nM, and 341 nM, respectively. In contrast, PM534 (**2**) displayed markedly enhanced potency, with GI_50_ values of 3.56 nM (A549), 3.80 nM (HT29), 3.80 nM (MDA-MB-231), and 3.08 nM (PSN). Notably, the *Z* isomer of (*Z*)-PM534 retained comparable activity, with GI_50_ values of 3.80 nM, 4.27 nM, 4.51 nM, and 4.51 nM across the same cell lines. These results indicate that PM534 (**2**) is highly potent and less sensitive to stereochemical variation at the oxime moiety, in contrast to PM742 (**1**).

## 3. Materials and Methods

### 3.1. General Experimental Procedures

Dry solvents were purchased and used without any extra processing. All reagents were used as purchased without further purification unless otherwise stated. All reactions were performed under an atmosphere of nitrogen in flame-dried or oven-dried glassware. Routine monitoring of reactions was performed using silica gel TLC plates (Merck 60 F254, Merck KGaA, Darmstadt, Germany). Spots were visualized by UV and/or dipping the TLC plate into an ethanolic phosphomolybdic acid solution and heating with a hot plate. Flash chromatography was carried out on silica gel 60 (200–400 mesh). ^1^H and ^13^C NMR were recorded on a Varian Unity 300, 400 or 500 spectrometers (Varian, Inc., Palo Alto, CA, USA) at 300 MHz, 400 MHz or 500 MHz, and 75 MHz, 100 MHz or 125 MHz, respectively. Chemical shifts (δ) are reported in parts per millions (ppm) referenced to CHCl_3_ at 7.26 ppm for ^1^H and CDCl_3_ at 77.0 ppm for ^13^C and to CH_3_OH at 3.30 ppm for ^1^H and CD_3_OD at 49.0 ppm. Coupling constants are reported in Hertz (Hz), with the following abbreviations used: s = singlet, d = doublet, t = triplet, q = quartet, m = multiplet. When appropriate, the multiplicities are preceded with br, indicating that the signal was broad. Optical rotations were determined using a Jasco P-1020 polarimeter (JASCO Corporation, Tokyo, Japan) with a sodium lamp and are reported as follows: [α]^25^_D_ (*c* g/100mL, solvent). (+)-ESIMS were recorded using an Agilent 1100 Series (Agilent Technologies, Palo Alto, CA, USA) LC/MSD spectrometer. The enantiomeric excess (*ee*) was determined by chiral HPLC analysis using an Agilent 1200 Series HPLC System equipped with a chiral stationary phase column. (+)-HRMS (ESI-TOF) was recorded using an Agilent 6230 TOF LC/MS system (Agilent Technologies, Santa Clara, CA, USA).

### 3.2. Total Synthesis of PM742 (***1***)

#### 3.2.1. Synthesis of Intermediate **6**

Compound **3**: To a solution of *N*-Boc-D-Norvaline (20 g, 92.0 mmol, 100% *ee*) in 2-Me-THF (368 mL, 4 mL/mmol) under a nitrogen atmosphere at 23 °C was added 1,1′-carbonyldiimidazole (CDI) (15.7 g, 96.6 mmol, 1.05 equiv.). The reaction mixture was stirred for 2 h at 23 °C. A solution of 2,2,6-trimethyl-4*H*-1,3-dioxin-4-one (30.55 mL, 230 mmol, 2.5 equiv.) in 2-Me-THF (368 mL, 4 mL/mmol) was slowly added to a precooled dilution at −78 °C of LiHMDS (368 mL, 1.0 M in THF, 368 mmol, 4.0 equiv.) in 2-Me-THF (368 mL, 4 mL/mmol). The reaction mixture was stirred at −78 °C for 1 h. ZnCl_2_ (31.3 g, 230 mmol, 2.5 equiv.) was added in one portion, and the reaction mixture was stirred at −78 °C for 30 min. Finally, the solution of the intermediate previously prepared was added by cannula at −78 °C. The reaction mixture was stirred at −78 °C for 4 h. An aqueous saturated solution of NH_4_Cl was added, and the aqueous layers were extracted with EtOAc. The combined organic layers were dried over anhydrous Na_2_SO_4_, filtered and concentrated under vacuum. The crude obtained was purified by column chromatography (CH_2_Cl_2_:EtOAc, 9:1) to give pure **3** (12.9 g, 41% yield, 98.5% *ee*). ^1^H NMR (300 MHz, CDCl_3_) δ: 5.35 (s, 1H), 5.02 (d, *J* = 7.9 Hz, 1H), 4.27 (td, *J* = 7.9, 4.7 Hz, 1H), 3.43 (s, 2H), 1.77 (m, 2H), 1.68 (s, 6H), 1.58–1.28 (m, 2H), 1.43 (s, 9H), 0.94 (t, *J* = 7.2 Hz, 3H). ^13^C NMR (75 MHz, CDCl_3_) δ: 203.2, 164.5, 160.8, 155.7, 107.4, 97.1, 80.4, 59.8, 43.9, 33.0, 28.5, 25.2, 18.8, 13.9. (+)ESIMS: *m/z* 364.3 [M + Na]^+^. (+)-HRMS (ESI-TOF) *m*/*z*: [M + Na]^+^ Calcd for C_17_H_27_NNaO_6_ 364.1731; Found 364.1734. R_f_: 0.13 (Hex:EtOAc 4:1).

Compound **4**: Compound **3** (10.74 g, 31.5 mmol) was dissolved in toluene (315 mL, 10 mL/mmol) and heated in a bath at 130 °C for 30 min. Evaporation of the solvent under vacuum afforded **4** crude (8.91 g, 99.5% *ee*) which was used in the next step without further purification. ^1^H NMR (400 MHz, CDCl_3_) δ: 10.50 (s, 1H), 6.09 (d, *J* = 1.8 Hz, 1H), 5.56 (s, 1H), 5.23 (d, *J* = 8.5Hz, 1H), 4.36 (q, *J* = 7.8 Hz, 1H), 1.78 (s, 1H), 1.67 (s, 1H), 1.43 (s, 9H), 0.92 (t, *J* = 7.3 Hz, 3H). ^13^C NMR (75 MHz, CDCl_3_) δ: 171.6, 166.9, 165.2, 155.8, 129.2, 128.4, 125.5, 100.9, 90.9, 80.9, 53.0, 35.3, 28.5, 19.3, 13.8. (+)ESIMS: *m*/*z* 306.1 [M + Na]^+^. (+)-HRMS (ESI-TOF) *m*/*z*: [M + Na]^+^ Calcd for C_14_H_21_NNaO_5_ 306.1312; Found 306.1314. R_f_: 0.35 (CH_2_Cl_2_:MeOH 9:1). [α]^25^_D_ 101.6 (*c* 0.018, MeOH).

Compound **5**: To a solution of **4** (8.92 g, 31.46 mmol) in acetone (314.6 mL, 10 mL/mmol) under a nitrogen atmosphere at 23 °C was added potassium carbonate (21.74 g, 157.3 mmol, 5.0 equiv.) and dimethyl sulfate (14.9 mL, 157.3 mmol, 5.0 equiv.). The reaction mixture was stirred for 2 h at 23 °C, filtered over Celite^®^, washed with CH_2_Cl_2_, and the solvent was removed under vacuum. The crude obtained was purified by column chromatography (Hexane:EtOAc, from 9:1 to 7:3) to give **5** pure (5.39 g, 60% yield for two steps). ^1^H NMR (400 MHz, CDCl_3_) δ: 5.93 (s, 1H), 5.45–5.40 (m, 1H), 4.86 (s, 1H), 4.38 (d, *J* = 8.2 Hz, 1H), 3.80 (s, 3H), 1.82–1.72 (m, 1H), 1.43 (t, *J* = 0.6 Hz, 9H), 1.38–1.30 (m, 2H), 0.93 (t, *J* = 7.3 Hz, 3H). ^13^C NMR (75 MHz, CDCl_3_) δ: 171.3, 164.6, 163.7, 155.1, 103.3, 100.0, 88.5, 56.2, 53.6, 52.7, 35.4, 29.9, 28.5, 19.3, 13.8. (+)ESIMS: *m*/*z* 320.0 [M + Na]^+^. (+)-HRMS (ESI-TOF) *m*/*z*: [M + Na]^+^ Calcd for C_15_H_23_NNaO_5_ 320.1468; Found 320.1471. R_f_: 0.3 (Hex:EtOAc 6:4). [α]^25^_D_ 92.2 (*c* 0.503, MeOH).

Compound **6**: To a solution of **5** (5.39 g, 18.1 mmol) in CH_2_Cl_2_ (202 mL, 37.5 mL/g) at 23 °C trifluoroacetic acid was added (59.3 mL, 11 mL/g). The reaction mixture was stirred for 1.5 h at 23 °C. Evaporation of the solvent under vacuum gave **6** crude, which was used in the next step without further purification. ^1^H NMR (300 MHz, CDCl_3_) δ: 6.16 (s, 1H), 5.54 (s, 1H), 4.13 (t, *J* = 7.5 Hz, 1H), 3.84 (s, 3H), 1.92 (q, *J* = 7.7 Hz, 2H), 1.29 (m, 2H), 0.93 (t, *J* = 7.3 Hz, 3H), 0.87 (m, 2H). ^13^C NMR (75 MHz, CDCl_3_) δ: 171.5, 165.4, 157.5, 141.6, 117.7, 104.0, 103.3, 89.8, 56.7, 53.1, 33.2, 29.9, 18.8, 13.3. (+)-HRMS (ESI-TOF) *m*/*z*: [M + H]^+^ Calcd for C_10_H_16_NO_3_ 198.1125; Found 198.1119. [α]^25^_D_ − 18.3 (*c* 0.497, MeOH).

#### 3.2.2. Final Steps

Compound **7**: 2-Methyl-L-cysteine hydrochloride (13 g, 75.74 mmol) was dissolved in the minimum quantity of H_2_O, cooled at 0 °C and basified with an aqueous saturated solution of NaHCO_3_ until pH 8. Evaporation of the solvent under vacuum afforded the corresponding sodium salt, which was dissolved in an aqueous saturated solution of NaHCO_3_ (151 mL, 2 mL/mmol). The aqueous solution was cooled to 0 °C and was added to DMF (151 mL, 2 mL/mmol) and 2,2-diethoxypropanenitrile (20 mL, 128 mmol, 1.7 equiv.). The reaction mixture was stirred overnight at 23 °C. After cooling at 0 °C, HCl 0.5 M was added until pH 2. The aqueous layer was extracted with a mixture of 50:50 Hex:EtOAc (x3). The combined organic layers were dried over anhydrous Na_2_SO_4_, filtered, and concentrated under vacuum to afford crude **7** (11.39 g, 57% yield, 99.2% *ee*) which was used in the next step without further purification. ^1^H NMR (300 MHz, CDCl_3_) δ: 3.72 (d, *J* = 11.6 Hz, 1H), 3.60–3.47 (m, 4H), 3.16 (d, *J* = 11.6 Hz, 1H), 1.59 (d, *J* = 1.9 Hz, 6H), 1.20 (t, *J* = 7.1, 6H). ^13^C NMR (75 MHz, CDCl_3_) δ: 175.6, 163.3, 100.5, 84.5, 57.9, 57.9, 40.7, 24.2, 23.9, 15.4. (+)-HRMS (ESI-TOF) *m*/*z*: [M + Na]^+^ Calcd for C_11_H_19_NNaO_4_S 284.0927; Found 284.0924. [α]^25^_D_ − 4.4 (*c* 0.098, MeOH).

Compound **8**: To a solution of **6** (6.76 g, 21.7 mmol) and **7** (5.68 g, 21.7 mmol) in CH_2_Cl_2_ (152 mL) was sequentially added at 23 °C *O*-(7-azabenzotriazoI-1-yl)-*N*,*N*,*N*′,*N*′-tetramethyluronium hexafluorophosphate (HATU) (17.02 g, 44.7 mmol, 2.06 equiv.), 1-hydroxy-7-azabenzotriazole (HOAt) (6.2 g, 45.1 mmol, 2.08 equiv.), and *N*,*N*-diisopropylethylamine (16.24 mL, 93 mmol, 4.29 equiv.). The reaction mixture was stirred for 15 h at 23 °C, diluted with CH_2_Cl_2_ and washed with an aqueous saturated solution of NaHCO_3_, HCl 0.5 M, and an aqueous saturated solution of NaCl. The combined organic layers were dried over anhydrous Na_2_SO_4_, filtered, and concentrated under vacuum. The obtained crude was purified by column chromatography (Hex:EtOAc, from 8:2 to 6:4) to obtain pure **8** (8.5 g, 89% yield for two steps). ^1^H NMR (400 MHz, CDCl_3_) δ: 7.02 (d, *J* = 9.0 Hz, 1H), 5.96–5.70 (m, 1H), 5.45–5.33 (m, 1H), 4.72 (td, *J* = 8.5, 6.2 Hz, 1H), 3.77 (s, 3H), 3.67–3.43 (m, 5H), 3.15 (d, *J* = 11.7 Hz, 1H), 1.87 (ddt, *J* = 13.1, 9.7, 6.4 Hz, 1H), 1.71 (ddd, *J* = 9.6, 8.3, 5.5 Hz, 2H), 1.61 (s, 3H), 1.53 (s, 3H), 1.44–1.28 (m, 2H), 1.22 (q, *J* = 7.2 Hz, 6H), 0.94 (t, *J* = 7.3 Hz, 3H). ^13^C NMR (75 MHz, CDCl_3_) δ: 177.1, 174.7, 171.0, 164.1, 163.2, 100.5, 99.8, 88.6, 85.4, 58.0, 56.1, 51.0, 40.6, 34.9, 25.5, 24.0, 19.3, 15.4, 13.8. (+)ESIMS: *m*/*z* 463.3 [M + Na]^+^. (+)-HRMS (ESI-TOF) *m*/*z*: [M + Na]^+^ Calcd for C_21_H_32_N_2_NaO_6_S 463.1873; Found 463.1875. R_f_: 0.29 (Hex:EtOAc 1:1). [α]^25^_D_ 51.1 (*c* 0.518, MeOH).

Compound **9**: Over **8** (4.25 g, 9.6 mmol) was added at 23 °C pentane (255 mL, 60 mL/g) and formic acid (170 mL, 40 mL/g). The reaction mixture was stirred vigorously for 2 h at 23 °C. The solvent was removed under vacuum. The obtained crude was purified by column chromatography (CH_2_Cl_2_:EtOAc from 9:1 to 8:2) to obtain pure **9** (4.25 g, 60% yield). ^1^H NMR (400 MHz, CDCl_3_) δ: 7.01 (d, *J* = 8.9 Hz, 1H), 5.91 (dd, *J* = 2.2, 0.4 Hz, 1H), 5.42 (t, *J* = 2.1 Hz, 1H), 4.74 (q, *J* = 7.8 Hz, 1H), 3.82–3.75 (m, 3H), 3.63 (dd, *J* = 12.0, 2.0 Hz, 1H), 3.28 (dd, *J* = 11.9, 0.9 Hz, 1H), 2.56 (d, *J* = 0.9 Hz, 3H), 1.95–1.73 (m, 1H), 1.54 (d, *J* = 2.0 Hz, 3H), 1.46–1.29 (m, 1H), 0.96 (td, *J* = 7.3, 1.7 Hz, 3H). ^13^C NMR (75 MHz, CDCl_3_) δ: 193.1, 173.2, 170.8, 170.4, 164.0, 162.0, 100.3, 88.6, 86.1, 56.0, 51.1, 40.1, 34.8, 26.3, 24.5, 19.0, 13.5. (+)ESIMS: *m*/*z* 367.1 [M + H]^+^, 389.1 [M + Na]^+^. (+)-HRMS (ESI-TOF) *m*/*z*: [M + Na]^+^ Calcd for C_17_H_22_N_2_NaO_5_S 389.1142; Found 389.1157. [α]^25^_D_ 67.3 (*c* 0.504, MeOH).

Compound PM742 (**1**): To a solution of **9** (4.25 g, 11.6 mmol) in ethanol (127.6 mL, 11 mL/mmol) and H_2_O (127.6 mL, 11 mL/mmol) was added at 23 °C hydroxylamine hydrochloride (5.96 g, 84.7 mmol, 7.4 equiv.) and sodium acetate (4.28 g, 52.2 mmol, 4.5 equiv.). The reaction mixture was stirred for 24 h at 23 °C. The solvent was removed under vacuum, and the residue obtained was dissolved in H_2_O and extracted with EtOAc. The combined organic layers were dried over anhydrous Na_2_SO_4_, filtered, and concentrated under vacuum. The obtained crude was purified by semipreparative HPLC (X-Bridge Prep C18, 5 μm, 19 × 150 mm, isocratic H_2_O:CH_3_CN (62:38), flow: 15 mL/min, UV detection) to yield (*Z*)-PM742 (320 mg, 7% yield, retention time: 6.0 min) and PM742 (**1**) (2.72 g, 64% yield, retention time: 9.3 min). The synthetic PM742 displayed physical, spectroscopic (^1^H, ^13^C NMR, and MS), and biological properties equivalent to those reported for natural PM742.

PM742: ^1^H NMR (500 MHz, CD_3_OD) δ: 6.08 (dd, *J* = 2.2, 0.7 Hz, 1H), 5.55 (d, *J* = 2.2 Hz, 1H), 4.72 (dd, *J* = 9.4, 5.4 Hz, 1H), 3.84 (s, 3H), 3.59 (d, *J* = 11.7 Hz, 1H), 3.22 (d, *J* = 11.6 Hz, 1H), 2.17 (s, 3H), 1.87 (dddd, *J* = 13.7, 9.6, 6.6, 5.4 Hz, 1H), 1.82–1.69 (m, 1H), 1.55 (s, 3H), 1.53–1.32 (m, 2H), 0.98 (t, *J* = 7.4 Hz, 3H). ^13^C NMR (125 MHz, CD_3_OD) δ: 176.5, 173.3, 170.2, 166.5, 165.1, 152.8, 100.7, 88.8, 85.5, 56.9, 52.1, 52.0, 40.5, 35.1, 35.1, 24.8, 20.1, 13.7, 10.8. (+)ESIMS: *m*/*z* 382.3 [M + H]^+^, 404.1 [M + Na]^+^. R_f_: 0.36 (Hex:EtOAc 1:1).

(*Z*)-PM742: ^1^H NMR (500 MHz, CD_3_OD) δ: 6.08 (dd, *J* = 2.2, 0.7 Hz, 1H), 5.55 (d, *J* = 2.2 Hz, 1H), 4.72 (dd, *J* = 9.4, 5.4 Hz, 1H), 3.84 (s, 3H), 3.59 (d, *J* = 11.7 Hz, 1H), 3.22 (d, *J* = 11.6 Hz, 1H), 2.17 (s, 3H), 1.87 (dddd, *J* = 13.7, 9.6, 6.6, 5.4 Hz, 1H), 1.82–1.69 (m, 1H), 1.55 (s, 3H), 1.53–1.32 (m, 2H), 0.98 (t, *J* = 7.4 Hz, 3H). ^13^C NMR (75 MHz, CDCl_3_) δ: 173.5, 171.0, 164.6, 164.5, 162.4, 147.2, 100.1, 88.5, 83.6, 56.0, 51.0, 40.9, 35.0, 24.8, 19.2, 19.0, 13.5.

### 3.3. Synthesis of PM534 (***2***)

Compound **10**: To a solution of **4** (9.9 g, 34.94 mmol) in DMF (800 mL) was added potassium carbonate (9.66 g, 69.89 mmol, 2 equiv.) at 23 °C. The reaction mixture was stirred for 30 min at 23 °C and cyclopropylmethyl bromide (3.7 mL, 38.44 mmol, 1 equiv.) was added at 23 °C. The reaction mixture was stirred overnight at 60 °C. The reaction mixture was concentrated under vacuum, diluted with EtOAc, filtered over Celite^®^ and washed with EtOAc. The crude obtained was purified in an automatic system for flash chromatography (SiO_2_, Hex:EtOAc 70:30) to yield **10** pure (10.13 g, 86% yield, 99.7% *ee*). ^1^H NMR (400 MHz, CDCl_3_) δ: 5.95 (d, *J* = 2.2 Hz, 1H), 5.34 (d, *J* = 2.2 Hz, 1H), 4.91 (d, *J* = 8.9 Hz, 1H), 4.37 (q, *J* = 7.6 Hz, 1H), 3.75 (dd, *J* = 7.1, 1.3 Hz, 2H), 1.77 (ddt, *J* = 13.3, 9.5, 6.5 Hz, 1H), 1.69–1.53 (m, 1H), 1.41 (s, 9H), 1.46–1.13 (m, 2H), 0.91 (t, *J* = 7.3 Hz, 3H), 0.72–0.59 (m, 2H), 0.39–0.26 (m, 2H). ^13^C NMR (100 MHz, CDCl_3_) δ: 170.2, 164.5, 163.5, 154.9, 99.9, 88.5, 80.0, 73.7, 52.5, 35.1, 28.3, 19.0, 13.5, 9.4, 3.3 (x2). (+)ESIMS: *m*/*z* 360.2 [M + Na]^+^. HRMS (ESI-TOF) *m*/*z*: [M + Na]^+^ Calcd for C_18_H_27_NNaO_5_ 360.1781; Found 360.1789. R_f_: 0.32 (Hex:EtOAc 7:3). [α]^25^_D_ 82.1 (*c* 0.046, MeOH).

Compound **11**: A solution of **10** (8.9 g, 26.44 mmol) in CH_2_Cl_2_ (334 mL) and trifluoroacetic acid (98 mL) was stirred at 23 °C for 2 h and then evaporated to dryness. The crude was evaporated three times with toluene to remove trifluoroacetic acid. The crude containing **11** (13.9 g, >100% yield) was used in the next step without further purification. ^1^H NMR (300 MHz, CDCl_3_) δ: 8.51 (s, 2H), 6.18 (d, *J* = 1.8 Hz, 1H), 5.48 (s, 1H), 4.13 (t, *J* = 7.3 Hz, 1H), 3.80 (d, *J* = 7.2 Hz,2H), 1.91 (q, *J* = 7.6 Hz, 2H), 1.38–1.18 (m, 2H), 0.92 (t, *J* = 7.3 Hz, 3H), 0.72–0.62 (m, 2H), 0.39–0.30 (m, 2H). ^13^C NMR (75 MHz, CDCl_3_) δ: 170.6, 157.2, 104.2, 89.8, 74.7, 52.9, 32.8, 18.5, 13.1, 9.2, 3.4, 3.3. HRMS (ESI-TOF) *m*/*z*: [M + Na]^+^ Calcd for C_13_H_19_NNaO_3_ 260.1257; Found 260.1261. [α]^25^_D_ − 89.0 (*c* 0.037, MeOH).

Compound **12**: To a solution of **11** (9.28 g, 26.41 mmol) and **7** (6.90 g, 26.41 mmol) in CH_2_Cl_2_ (180 mL) was sequentially added *O*-(7-azabenzotriazol-1-yl)-*N*,*N*,*N*′,*N*′-tetramethyluronium hexafluorophosphate (HATU) (10.04 g, 26.41 mmol, 1 equiv.), 1-hydroxy-7-azabenzotriazole (HOAt) (3.62 g, 26.41 mmol, 1 equiv.), and *N*,*N*-diisopropylethylamine (18.4 mL, 105.66 mmol, 4 equiv.) at 23 °C. The reaction mixture was stirred overnight at 23 °C, diluted with CH_2_Cl_2_ and washed HCl 0.5 M. The aqueous layer was extracted with CH_2_Cl_2_ (x2). The combined organic layers were dried over anhydrous Na_2_SO_4_, filtered and concentrated under vacuum. The obtained crude was purified in an automatic system for flash chromatography (SiO_2_, Hex:EtOAc 50:50) to obtain pure **12** (12.1 g, 95% yield for two steps, 100% *ee*). ^1^H NMR (400 MHz, CDCl_3_) δ: 7.03 (d, *J* = 8.9 Hz, 1H), 5.85 (dd, *J* = 2.2, 0.5 Hz, 1H), 5.31 (d, *J* = 2.2 Hz, 1H), 4.71 (td, *J* = 8.5, 6.2 Hz, 1H), 3.80–3.67 (m, 2H), 3.61 (d, *J* = 11.8 Hz, 1H), 3.61–3.42 (m, 4H), 3.14 (d, *J* = 11.7 Hz, 1H), 1.86 (ddt, *J* = 13.7, 9.5, 6.3 Hz, 1H), 1.75–1.62 (m, 1H), 1.60 (s, 3H), 1.52 (s, 3H), 1.45–1.15 (m, 2H), 1.22 (t, *J* = 7.1 Hz, 3H), 1.20 (t, *J* = 7.1 Hz, 3H), 0.92 (t, *J* = 7.3 Hz, 3H), 0.74–0.58 (m, 2H), 0.38–0.27 (m, 2H). ^13^C NMR (100 MHz, CDCl_3_) δ: 176.8, 174.4, 170.0, 163.9, 162.9, 100.2, 99.8, 88.6, 85.2, 73.7, 57.7, 57.6, 50.8, 40.3, 34.7, 25.3, 23.7, 19.0, 15.2, 13.5, 9.4, 3.3 (x2). (+)ESIMS: *m*/*z* 503.3 [M + Na]^+^. (+)-HRMS (ESI-TOF) *m*/*z*: [M + Na]^+^ Calcd for C_24_H_36_N_2_NaO_6_S 503.2186; Found 503.2203. R_f_: 0.49 (Hex:EtOAc 1:1). [α]^25^_D_ 47.3 (*c* 0.424, MeOH).

Compound **13**: Over **12** (9.0 g, 18.73 mmol) was added at 23 °C pentane (460 mL) and formic acid (315 mL). The reaction mixture was stirred vigorously for 2 h at 23 °C and the volatiles were evaporated under vacuum. The obtained crude was evaporated a few times with a mixture of CH_2_Cl_2_:toluene to eliminate formic acid to give crude **13** (7.61 g, 100% yield), which was used in the next step without further purification. ^1^H NMR (400 MHz, CDCl_3_) δ: 7.05 (d, *J* = 8.9 Hz, 1H), 5.94 (d, *J* = 2.2 Hz, 1H), 5.37 (d, *J* = 2.3 Hz, 1H), 4.73 (q, *J* = 7.9 Hz, 1H), 3.76 (dd, *J* = 7.1,1.9 Hz, 2H), 3.61 (d, *J* = 11.9 Hz, 1H), 3.27 (d, *J* = 12.0 Hz, 1H), 2.55 (s, 3H), 1.94–1.73 (m, 1H), 1.66–1.53 (m, 1H), 1.53 (s, 3H), 1.52–1.16 (m, 2H), 0.95 (t, *J* = 7.4 Hz, 3H), 0.70–0.61 (m, 2H), 0.33 (t, *J* = 5.2 Hz, 2H). ^13^C NMR (100 MHz, CDCl_3_) δ: 93.2, 173.2, 170.4, 164.3, 163.0, 161.8, 100.8, 88.9, 86.0, 73.9, 51.0, 40.1, 34.8, 26.3, 24.5, 19.0, 13.5, 9.3, 3.4, 3.3. (+)ESIMS: *m*/*z* 407.1 [M + H]^+^, 429.2 [M + Na]^+^. HRMS (ESI-TOF) *m*/*z*: [M + H]^+^ Calcd for C_20_H_27_N_2_O_5_S 407.1635; Found 407.1635. R_f_: 0.47 (Hex:EtOAc 1:1). [α]^25^_D_ 56.2 (*c* 0.019, MeOH).

Compound PM534 (**2**): To a solution of crude **13** (33 mg, 0.08 mmol) in EtOH (0.9 mL) and H_2_O (0.9 mL) was added hydroxylamine hydrochloride (42 mg, 0.6 mmol, 7.5 equiv.) and sodium acetate (30 mg, 0.36 mmol, 4.5 equiv.) at 23 °C. The reaction mixture was stirred for 24 h at 23 °C and concentrated under vacuum. The residue obtained was diluted with an aqueous saturated solution of NaCl and extracted with EtOAc (x3). The combined organic layers were dried over anhydrous Na_2_SO_4_, filtered, and concentrated under vacuum. The obtained crude was purified by flash chromatography on silica gel (Hex:EtOAc, from 100:0 to 40:60) to afford PM534 (**2**) (15 mg, 44% yield, 100% *ee*) and (*Z*)-PM534 (3 mg, 9% yield).

PM534: ^1^H NMR (400 MHz, CD_3_OD) δ: 7.85 (d, *J* = 8.6 Hz, 1H), 6.05 (s, 1H), 5.48 (s, 1H), 4.74 (q, *J* = 6.8, 5.0 Hz, 1H), 3.86 (d, *J* = 7.2 Hz, 2H), 3.56 (d, *J* = 11.5 Hz, 1H), 3.17 (d, *J* = 11.5 Hz, 1H), 2.19 (s, 3H), 1.96–1.72 (m, 2H), 1.52 (s, 3H), 1.43 (ddd, *J* = 29.8, 14.7, 7.4 Hz, 4H), 1.31–1.16 (m, 1H), 0.99 (t, *J* = 7.4 Hz, 3H), 0.63 (d, *J* = 7.6 Hz, 2H), 0.35 (d, *J* = 5.0 Hz, 2H). ^13^C NMR (100 MHz, CD_3_OD) δ: 175.1, 171.2, 168.8, 165.3, 163.8, 151.5, 126.4, 99.7, 87.9, 84.2, 74.0, 50.7, 39.1, 33.9, 23.6, 18.8, 12.5, 9.6, 9.0, 2.3. (+)ESIMS: *m*/*z* 422.1 [M + H]^+^, 444.2 [M + Na]^+^. HRMS (ESI-TOF) *m*/*z*: [M + Na]^+^ Calcd for C_20_H_27_N_3_NaO_5_S 444.1564; Found 444.1563. R_f_: 0.42 (Hex:EtOAc 1:1). [α]^25^_D_ 55.0 (*c* 0.022, MeOH).

(*Z*)-PM534: ^1^H NMR (400 MHz, CD_3_OD) δ: 7.84 (d, *J* = 8.5 Hz, 0H), 6.09 (dd, *J* = 0.7, 2.2 Hz, 1H), 5.49 (d, *J* = 2.2 Hz, 1H), 4.72 (dd, *J* = 5.5, 9.4 Hz, 1H), 3.87 (d, *J* = 7.2 Hz, 2H), 3.59 (d, *J* = 11.7 Hz, 1H), 3.30–3.14 (m, 1H), 2.17 (d, *J* = 8.9 Hz, 7H), 2.02–1.68 (m, 3H), 1.59–1.32 (m, 5H), 1.30–1.13 (m, 1H), 1.03–0.90 (m, 3H), 0.69–0.58 (m, 2H), 0.40–0.31 (m, 2H). ^13^C NMR (100 MHz, CDCl_3_) δ: 173.8, 170.1, 168.4, 164.3, 162.3, 153.3, 100.5, 88.9, 84.2, 73.8, 51.1, 39.9, 34.8, 24.7, 19.1, 13.6, 11.3, 9.4, 3.4 (x2). (+)ESIMS: *m*/*z* 422.1 [M + H]^+^. (+)-HRMS (ESI-TOF) *m*/*z*: [M + H]^+^ Calcd for C_20_H_28_N_3_O_5_S 422.1744; Found 422.1758. [α]^25^_D_ 73.7 (*c* 0.428, MeOH).

### 3.4. Bioassay for the Detection of Antitumor Activity

The aim of this assay is to evaluate the in vitro cytostatic (ability to delay or arrest tumor cell growth) or cytotoxic (ability to kill tumor cells) activity of the samples being tested. A colorimetric assay, using the sulforhodamine B (SRB) reaction has been adapted to provide a quantitative measurement of cell growth and viability [9,10]. This form of assay employs 96-well cell culture microplates. All the cell lines used in this study were obtained from the American Type Culture Collection (ATCC), unless otherwise indicated, and derived from different types of human cancer: A-549 (ATCC CCL-185), lung carcinoma; HT-29 (ATCC HTB-38), colorectal carcinoma, MDA-MB-231 (ATCC HTB-26), breast adenocarcinoma and PSN-1, pancreatic adenocarcinoma [11]. Cell lines were maintained in Dulbecco’s Modified Eagle Medium (DMEM) (for A-549, HT-29, and MDA-MB-231) or RPMI (for PSN-1) supplemented with 10% Fetal Bovine Serum (FBS), 2 mM L-glutamine, 100 U/mL penicillin, and 100 U/mL streptomycin, at 37 °C, 5% CO_2_ and 98% humidity. For the experiments, cells were harvested from subconfluent cultures using trypsinization and resuspended in a fresh medium before counting and plating.

Cells were seeded in 96-well microtiter plates at 5 × 10^3^ cells per well in aliquots of 150 μL and allowed to attach to the plate surface for 18 h (overnight) in a drug-free medium. After that, one control (untreated) plate of each cell line was fixed (as described below) and used for the time zero reference value. Culture plates were then treated with test compounds (50 μL aliquots of 4X concentrated compound stock solutions made in complete culture medium) using ten serial dilutions (concentrations ranging from 10 μg/mL to 0.000262 μg/mL) and triplicate cultures (final concentration of DMSO being 1%). After 72 h treatment, the antitumor effect was measured by using the SRB methodology: briefly, cells were washed twice with PBS, fixed for 15 min in 1% glutaraldehyde solution at room temperature, rinsed twice in PBS, and stained in 0.4% SRB solution for 30 min at room temperature. Cells were then rinsed several times with 1% acetic acid solution and air-dried at room temperature. SRB was then extracted in a 10 mM trizma base solution, and the absorbance was measured in an automated spectrophotometric plate reader at 490 nm. Effects on cell growth and survival were estimated by applying the NCI algorithm [12]. In this assay Doxorubicin and DMSO (solvent) were used as the positive and negative controls, respectively. Prism 3.03 from GraphPad was used for the statistical analysis of the cell growth inhibition results. Using the mean ± SD of triplicates, a dose–response curve was automatically generated using nonlinear regression analysis to a 4-parameter logistic curve. Three reference parameters were calculated (NCI algorithm) by automatic interpolation: GI_50_ = compound concentration that produces 50% cell growth inhibition, as compared to control cultures.

## 4. Conclusions

This work describes the first total synthesis of the marine natural product PM742 (**1**) through an efficient and convergent strategy that also enabled the preparation of a range of structurally related derivatives. The synthetic approach provides practical access to PM742 and facilitates further analog development. PM742 exhibited in vitro cytotoxic activity against human tumor cell lines, which motivated preliminary structure–activity relationship (SAR) studies. These efforts led to the identification of PM534 (**2**), a related analog with improved activity in the same assays. Preliminary studies suggest that PM534 may act through multiple mechanisms, including effects on tumor cell division and vasculature, supporting its further evaluation as a potential anticancer agent.

## Figures and Tables

**Table 1:** In vitro cytotoxic activity (GI_50_, nM).

	Lung A-549	Colon HT-29	Breast MDA-MB-231	Pancreas PSN-1
PM742 (**1**)	11.5	14.4	34.1	28.8
(*Z*)-PM742	144	152	204	341
PM534 (**2**)	3.56	3.80	3.80	3.08
(*Z*)-PM534	3.80	4.27	4.51	4.51

## Data Availability

The data are contained within the article or Supplementary Materials.

## Supplementary Materials

The following supporting information can be downloaded at: https://www.mdpi.com/article/10.3390/md24050167/s1, Figure S1: ^1^H NMR spectra of PM742 (**1**) synthetic vs. natural; Figures S2–S15: NMR spectra of the synthesis intermediates of PM742 (**1**); Figures S16–S19: NMR spectra of PM742 (**1**) and (*Z*)-PM742; Figure S20: ROESY spectrum of (*Z*)-PM742; Figures S21–S28: NMR spectra of the synthesis intermediates of PM534 (**2**); Figures S29–S32: NMR spectra of PM534 (**2**) and (*Z*)-P534.
