# Supplementary material for: Convergent Total Synthesis of PM742 and SAR-Guided Development of the Clinical Candidate PM534
Source: Mar Drugs. 2026 May 7;24(5):167. doi: 10.3390/md24050167 (PMC13208196; doi:10.3390/md24050167)

# Supplementary Material

## Convergent Total Synthesis of PM742 and SAR-Guided Development of the Clinical Candidate PM534

María Jesús Martín, Juan Hernando, Raquel Rodríguez-Acebes, Asier Gómez-SanJuan, Andrés Francesch, Simon Munt, Carmen Cuevas *

R&D, PharmaMar, S. A., Avenida de los Reyes, 1, 28770 - Colmenar Viejo, Madrid, Spain.

## Table of contents

**Figure S1.** $^{1}$H NMR spectra of PM742 (**1**) synthetic *vs* natural 4

**Figure S2.** $^{1}$H NMR spectrum of *tert*-Butyl (*R*)-(1-(2,2-dimethyl-4-oxo-4*H*-1,3-dioxin-6-yl)-2-oxohexan-3-yl)carbamate (**3**). (300 MHz, $CDCl_3$). 5

**Figure S3.** $^{13}$C NMR spectrum of *tert*-Butyl (*R*)-(1-(2,2-dimethyl-4-oxo-4*H*-1,3-dioxin-6-yl)-2-oxohexan-3-yl)carbamate (**3**). (75 MHz, $CDCl_3$). 6

**Figure S4.** $^{1}$H NMR spectrum of *tert*-Butyl (*R*)-(1-(4-hydroxy-2-oxo-2*H*-pyran-6-yl)butyl)carbamate (**4**). (400 MHz, $CDCl_3$). 7

**Figure S5.** $^{13}$C NMR spectrum of *tert*-Butyl (*R*)-(1-(4-hydroxy-2-oxo-2*H*-pyran-6-yl)butyl)carbamate (**4**). (75 MHz, $CDCl_3$). 8

**Figure S6.** $^{1}$H NMR spectrum of *tert*-Butyl (*R*)-(1-(4-methoxy-2-oxo-2*H*-pyran-6-yl)butyl)carbamate (**5**). (400 MHz, $CDCl_3$). 9

**Figure S7.** $^{13}$C NMR spectrum of *tert*-Butyl (*R*)-(1-(4-methoxy-2-oxo-2*H*-pyran-6-yl)butyl)carbamate (**5**). (75 MHz, $CDCl_3$). 10

**Figure S8.** $^{1}$H NMR spectrum of (*R*)-1-(4-Methoxy-2-oxo-2*H*-pyran-6-yl)butan-1-aminium 2,2,2-trifluoroacetate (**6**). (300 MHz, $CDCl_3$). 11

**Figure S9.** $^{13}$C NMR spectrum of (*R*)-1-(4-Methoxy-2-oxo-2*H*-pyran-6-yl)butan-1-aminium 2,2,2-trifluoroacetate (**6**). (75 MHz, $CDCl_3$). 12

**Figure S10.** $^{1}$H NMR spectrum of (*R*)-2-(1,1-Diethoxyethyl)-4-methyl-4,5-dihydrothiazole-4-carboxylic acid (**7**). (300 MHz, $CDCl_3$). 13

**Figure S11.** $^{13}$C NMR spectrum of (*R*)-2-(1,1-Diethoxyethyl)-4-methyl-4,5-dihydrothiazole-4-carboxylic acid (**7**). (75 MHz, $CDCl_3$). 14

**Figure S12.** $^{1}$H NMR spectrum of (*R*)-2-(1,1-Diethoxyethyl)-*N*-((*R*)-1-(4-methoxy-2-oxo-2*H*-pyran-6-yl)butyl)-4-methyl-4,5-dihydrothiazole-4-carboxamide (**8**). (400 MHz, $CDCl_3$). 15

**Figure S13.** $^{13}$C NMR spectrum of (*R*)-2-(1,1-Diethoxyethyl)-*N*-((*R*)-1-(4-methoxy-2-oxo-2*H*-pyran-6-yl)butyl)-4-methyl-4,5-dihydrothiazole-4-carboxamide (**8**). (75 MHz, $CDCl_3$). 16

**Figure S14.** $^{1}$H NMR spectrum of (*R*)-2-Acetyl-*N*-((*R*)-1-(4-methoxy-2-oxo-2*H*-pyran-6-yl)butyl)-4-methyl-4,5-dihydrothiazole-4-carboxamide (**9**). (400 MHz, $CDCl_3$). 17

**Figure S15.** $^{13}$C NMR spectrum of (*R*)-2-Acetyl-*N*-((*R*)-1-(4-methoxy-2-oxo-2*H*-pyran-6-yl)butyl)-4-methyl-4,5-dihydrothiazole-4-carboxamide (**9**). (75 MHz, $CDCl_3$). 18

**Figure S16.** $^{1}$H NMR spectrum of (*R*)-2-((*E*)-1-(Hydroxyimino)ethyl)-*N*-((*R*)-1-(4-methoxy-2-oxo-2*H*-pyran-6-yl)butyl)-4-methyl-4,5-dihydrothiazole-4-carboxamide (**PM742**). (500 MHz, $CD_3OD$). 19

**Figure S17.** $^{13}$C NMR spectrum of (*R*)-2-((*E*)-1-(Hydroxyimino)ethyl)-*N*-((*R*)-1-(4-methoxy-2-oxo-2*H*-pyran-6-yl)butyl)-4-methyl-4,5-dihydrothiazole-4-carboxamide (**PM742**). (125 MHz, $CD_3OD$). 20

**Figure S18.** $^{1}$H NMR spectrum of (*R*)-2-((*Z*)-1-(Hydroxyimino)ethyl)-*N*-((*R*)-1-(4-methoxy-2-oxo-2*H*-pyran-6-yl)butyl)-4-methyl-4,5-dihydrothiazole-4-carboxamide (*Z*-**PM742**). (500 MHz, $CD_3OD$). 21

**Figure S19.** $^{13}$C NMR spectrum of (*R*)-2-((*Z*)-1-(Hydroxyimino)ethyl)-*N*-((*R*)-1-(4-methoxy-2-oxo-2*H*-pyran-6-yl)butyl)-4-methyl-4,5-dihydrothiazole-4-carboxamide (*Z*-**PM742**). (75 MHz, $CDCl_3$). 22

**Figure S20.** ROESY spectrum of (*R*)-2-((*Z*)-1-(Hydroxyimino)ethyl)-*N*-((*R*)-1-(4-methoxy-2-oxo-2*H*-pyran-6-yl)butyl)-4-methyl-4,5-dihydrothiazole-4-carboxamide (*Z*-**PM742**). (500 MHz, $(CD_3)_2SO$). 23

**Figure S21.** $^{1}$H NMR spectrum of *tert*-Butyl (*R*)-(1-(4-(cyclopropylmethoxy)-2-oxo-2*H*-pyran-6-yl)butyl)carbamate (**10**). (400 MHz, $CDCl_3$). 24

**Figure S22.** $^{13}$C NMR spectrum of *tert*-Butyl (*R*)-(1-(4-(cyclopropylmethoxy)-2-oxo-2*H*-pyran-6-yl)butyl)carbamate (**10**). (100 MHz, $CDCl_3$). 25

**Figure S23.** $^{1}$H NMR spectrum of (*R*)-1-(4-(Cyclopropylmethoxy)-2-oxo-2*H*-pyran-6-yl)butan-1-aminium 2,2,2-trifluoroacetate (**11**). (300 MHz, $CDCl_3$). 26

**Figure S24.** $^{13}$C NMR spectrum of (*R*)-1-(4-(Cyclopropylmethoxy)-2-oxo-2*H*-pyran-6-yl)butan-1-aminium 2,2,2-trifluoroacetate (**11**). (75 MHz, $CDCl_3$). 27

**Figure S25.** $^{1}$H NMR spectrum of (*R*)-*N*-((*R*)-1-(4-(Cyclopropylmethoxy)-2-oxo-2*H*-pyran-6-yl)butyl)-2-(1,1-diethoxyethyl)-4-methyl-4,5-dihydrothiazole-4-carboxamide (**12**). (400 MHz, $CDCl_3$). 28

**Figure S26.** $^{13}$C NMR spectrum of (*R*)-*N*-((*R*)-1-(4-(Cyclopropylmethoxy)-2-oxo-2*H*-pyran-6-yl)butyl)-2-(1,1-diethoxyethyl)-4-methyl-4,5-dihydrothiazole-4-carboxamide (**12**). (100 MHz, $CDCl_3$). 29

**Figure S27.** $^{1}$H NMR spectrum of (*R*)-2-Acetyl-*N*-((*R*)-1-(4-(cyclopropylmethoxy)-2-oxo-2*H*-pyran-6-yl)butyl)-4-methyl-4,5-dihydrothiazole-4-carboxamide (**13**). (400 MHz, $CDCl_3$). 30

**Figure S28.** $^{13}$C NMR spectrum of (*R*)-2-Acetyl-*N*-((*R*)-1-(4-(cyclopropylmethoxy)-2-oxo-2*H*-pyran-6-yl)butyl)-4-methyl-4,5-dihydrothiazole-4-carboxamide (**13**). (100 MHz, $CDCl_3$). 31

**Figure S29.** $^{1}$H NMR spectrum of (*R*)-*N*-((*R*)-1-(4-(Cyclopropylmethoxy)-2-oxo-2*H*-pyran-6-yl)butyl)-2-((*E*)-1-(hydroxyimino)ethyl)-4-methyl-4,5-dihydrothiazole-4-carboxamide (**PM534**). (400 MHz, $CD_3OD$). 32

**Figure S30.** $^{13}$C NMR spectrum of (*R*)-*N*-((*R*)-1-(4-(Cyclopropylmethoxy)-2-oxo-2*H*-pyran-6-yl)butyl)-2-((*E*)-1-(hydroxyimino)ethyl)-4-methyl-4,5-dihydrothiazole-4-carboxamide (**PM534**). (100 MHz, $CDCl_3$). 33

**Figure S31.** $^{1}$H NMR spectrum of (*R*)-*N*-((*R*)-1-(4-(Cyclopropylmethoxy)-2-oxo-2*H*-pyran-6-yl)butyl)-2-((*Z*)-1-(hydroxyimino)ethyl)-4-methyl-4,5-dihydrothiazole-4-carboxamide (*Z*-**PM534**). (400 MHz, $CD_3OD$). 34

**Figure S32.** $^{13}$C NMR spectrum of (*R*)-*N*-((*R*)-1-(4-(Cyclopropylmethoxy)-2-oxo-2*H*-pyran-6-yl)butyl)-2-((*Z*)-1-(hydroxyimino)ethyl)-4-methyl-4,5-dihydrothiazole-4-carboxamide (*Z*-**PM534**). (100 MHz, $CDCl_3$). 35

**Figure S1.** $^{1}$H NMR spectra of PM742 (**1**) synthetic *vs* natural

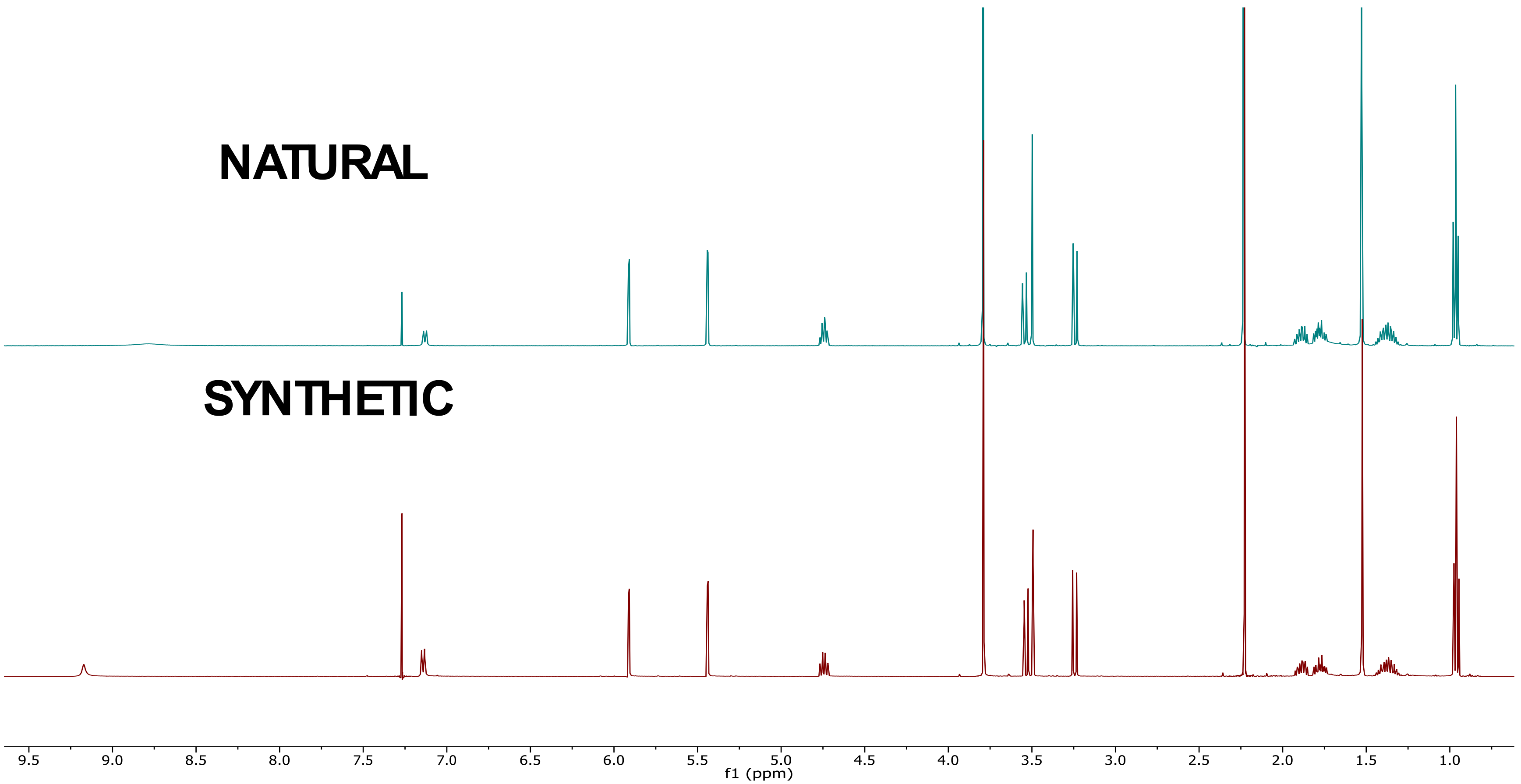

**Figure S2.** $^{1}$H NMR spectrum of *tert*-Butyl (*R*)-(1-(2,2-dimethyl-4-oxo-4*H*-1,3-dioxin-6-yl)-2-oxohexan-3-yl)carbamate (**3**). (300 MHz, $CDCl_3$).

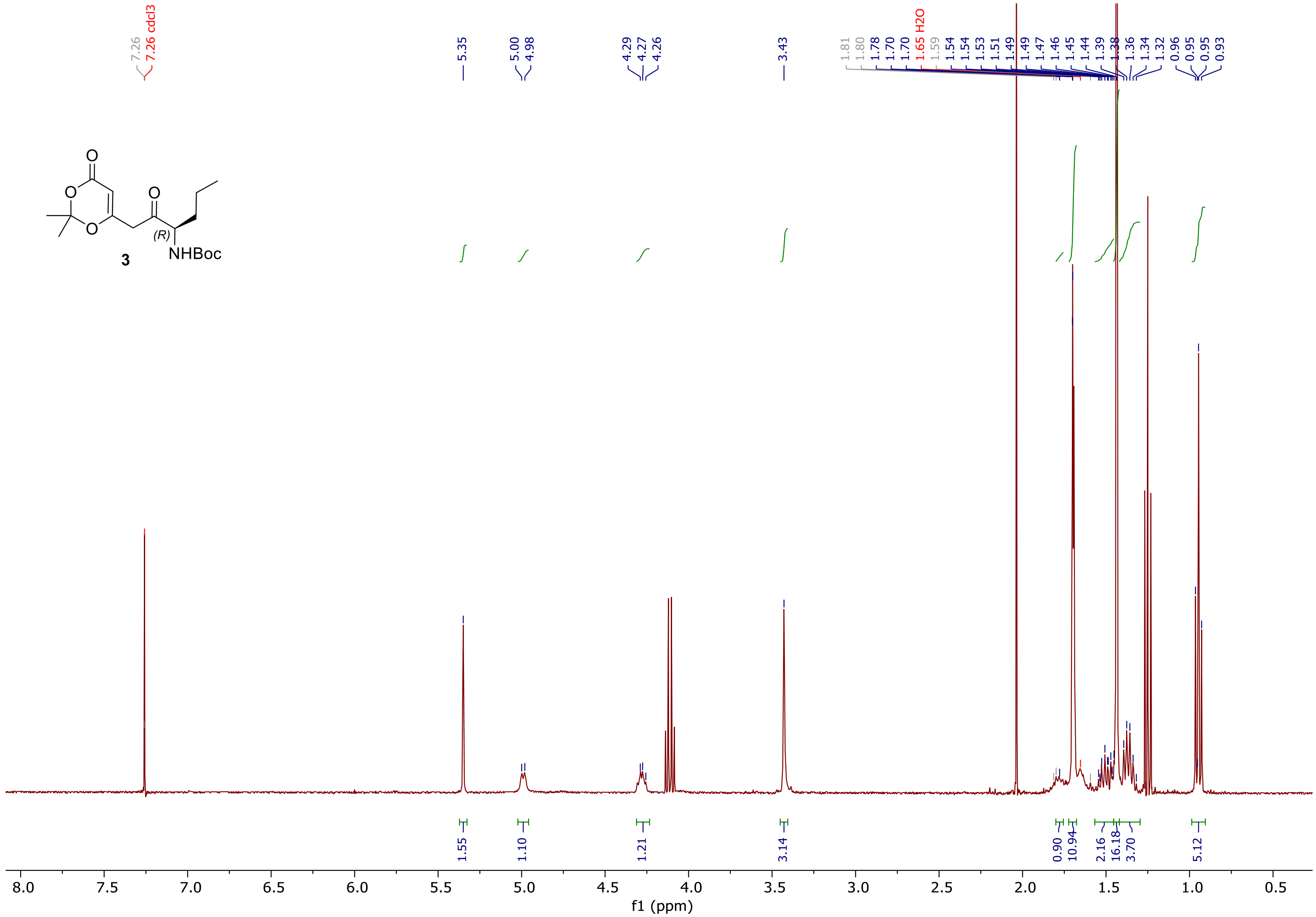

**Figure S3.** $^{13}C$ NMR spectrum of *tert*-Butyl (*R*)-(1-(2,2-dimethyl-4-oxo-4*H*-1,3-dioxin-6-yl)-2-oxohexan-3-yl)carbamate (**3**). (75 MHz, $CDCl_3$).

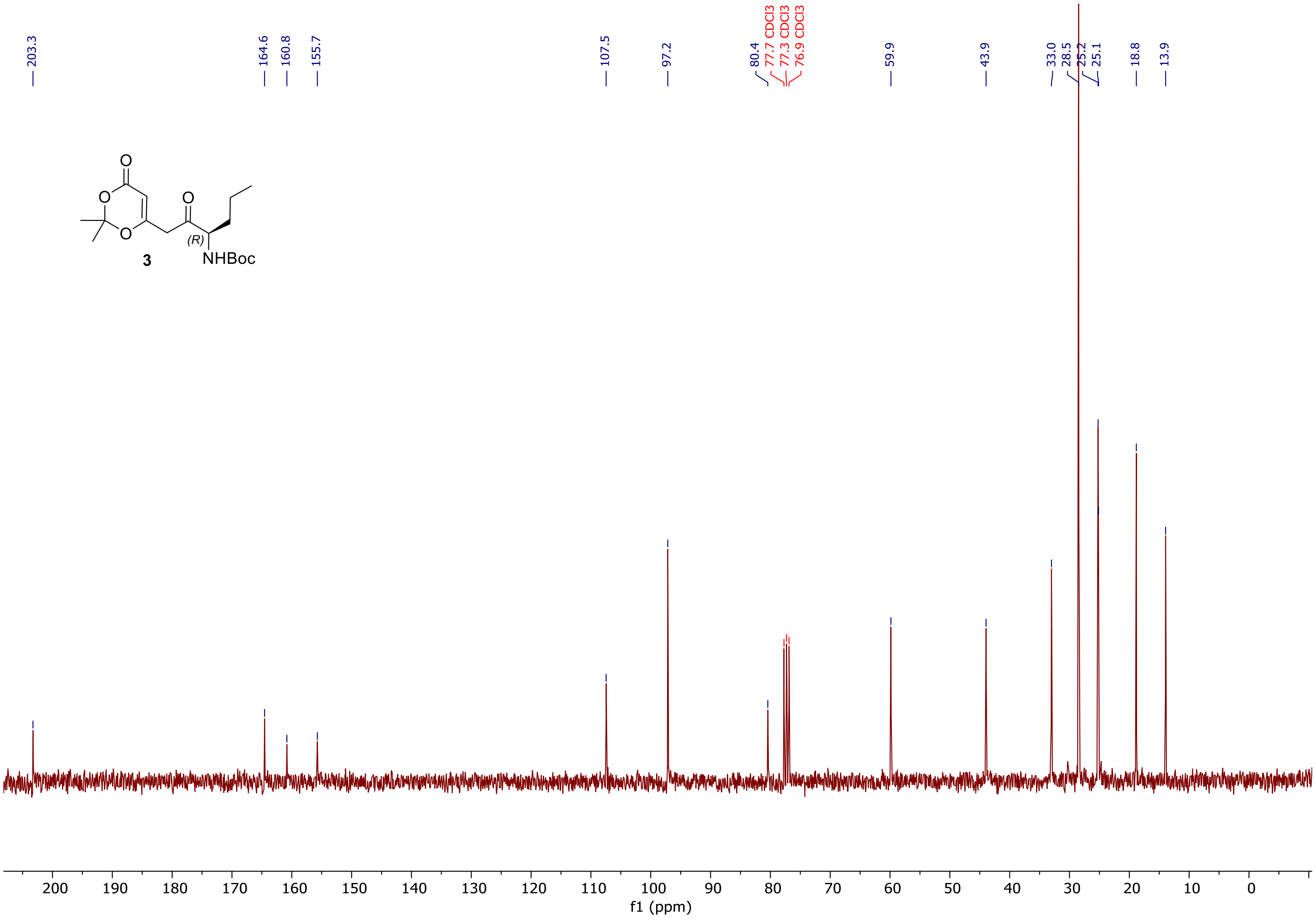

**Figure S4.** $^{1}$H NMR spectrum of *tert*-Butyl (*R*)-(1-(4-hydroxy-2-oxo-2*H*-pyran-6-yl)butyl)carbamate (**4**). (400 MHz, CDCl$_3$).

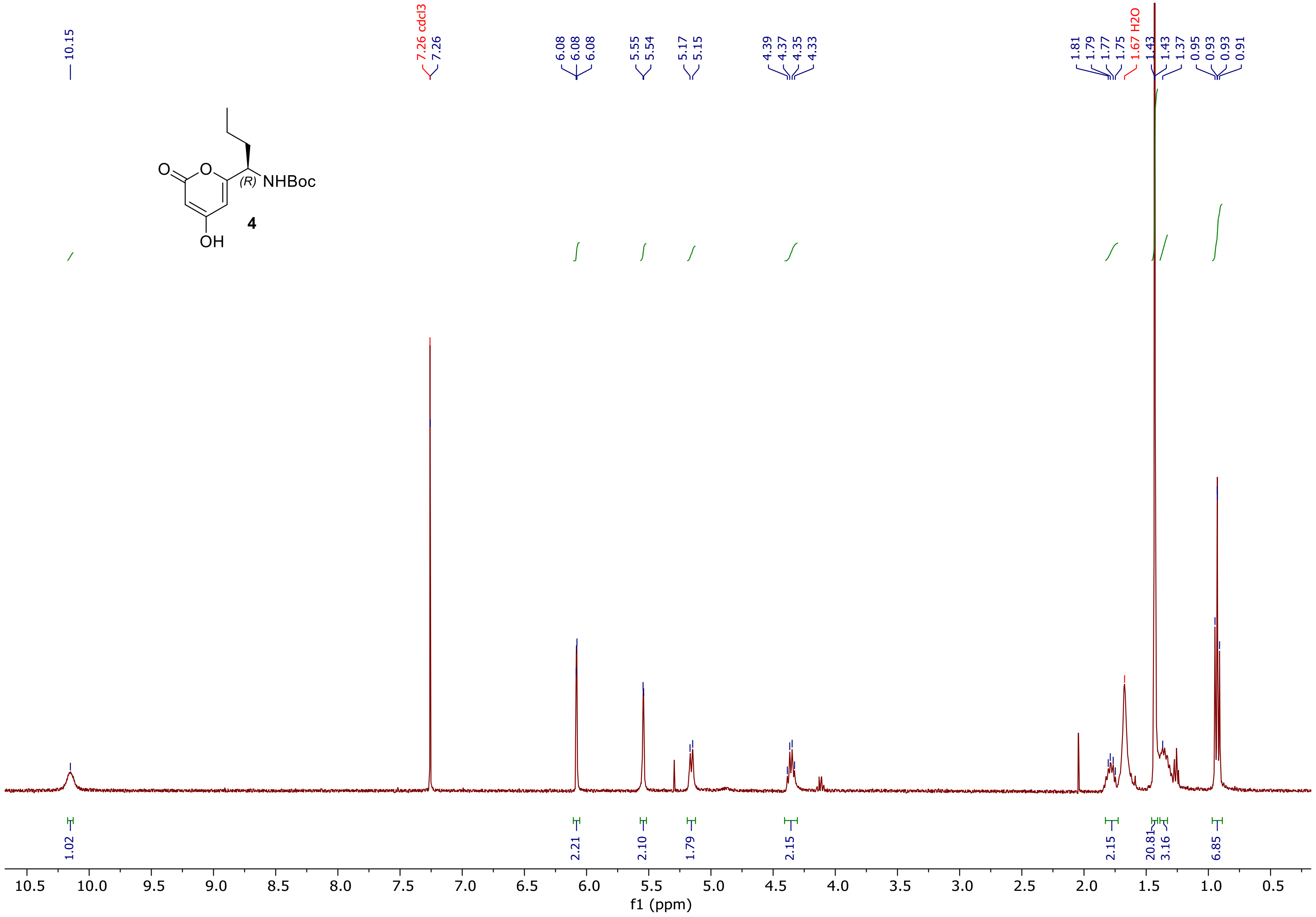

**Figure S5.** $^{13}C$ NMR spectrum of *tert*-Butyl (*R*)-(1-(4-hydroxy-2-oxo-2*H*-pyran-6-yl)butyl)carbamate (**4**). (75 MHz, $CDCl_3$).

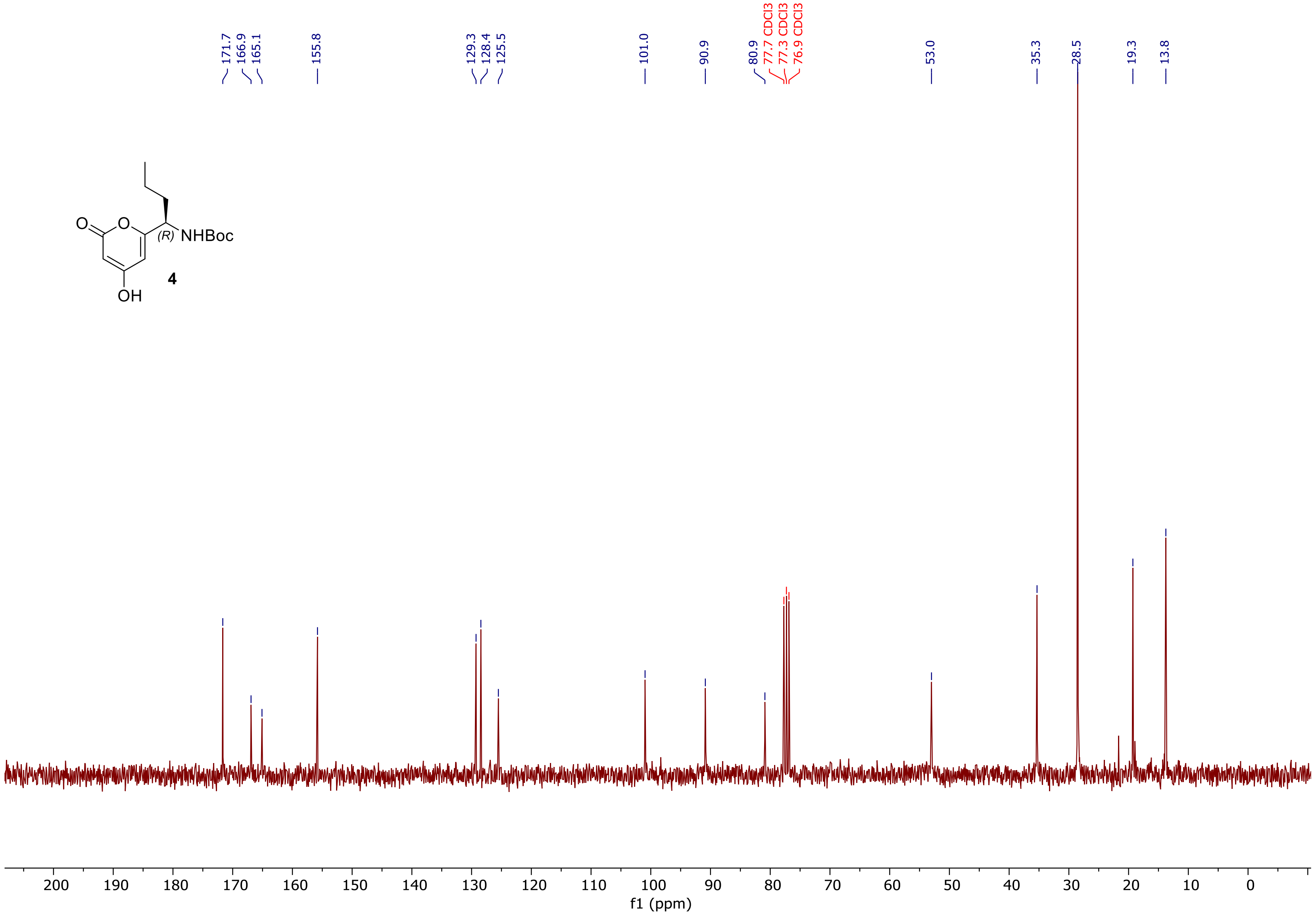

**Figure S6.** $^{1}H$ NMR spectrum of *tert*-Butyl (*R*)-(1-(4-methoxy-2-oxo-2*H*-pyran-6-yl)butyl)carbamate (**5**). (400 MHz, $CDCl_3$).

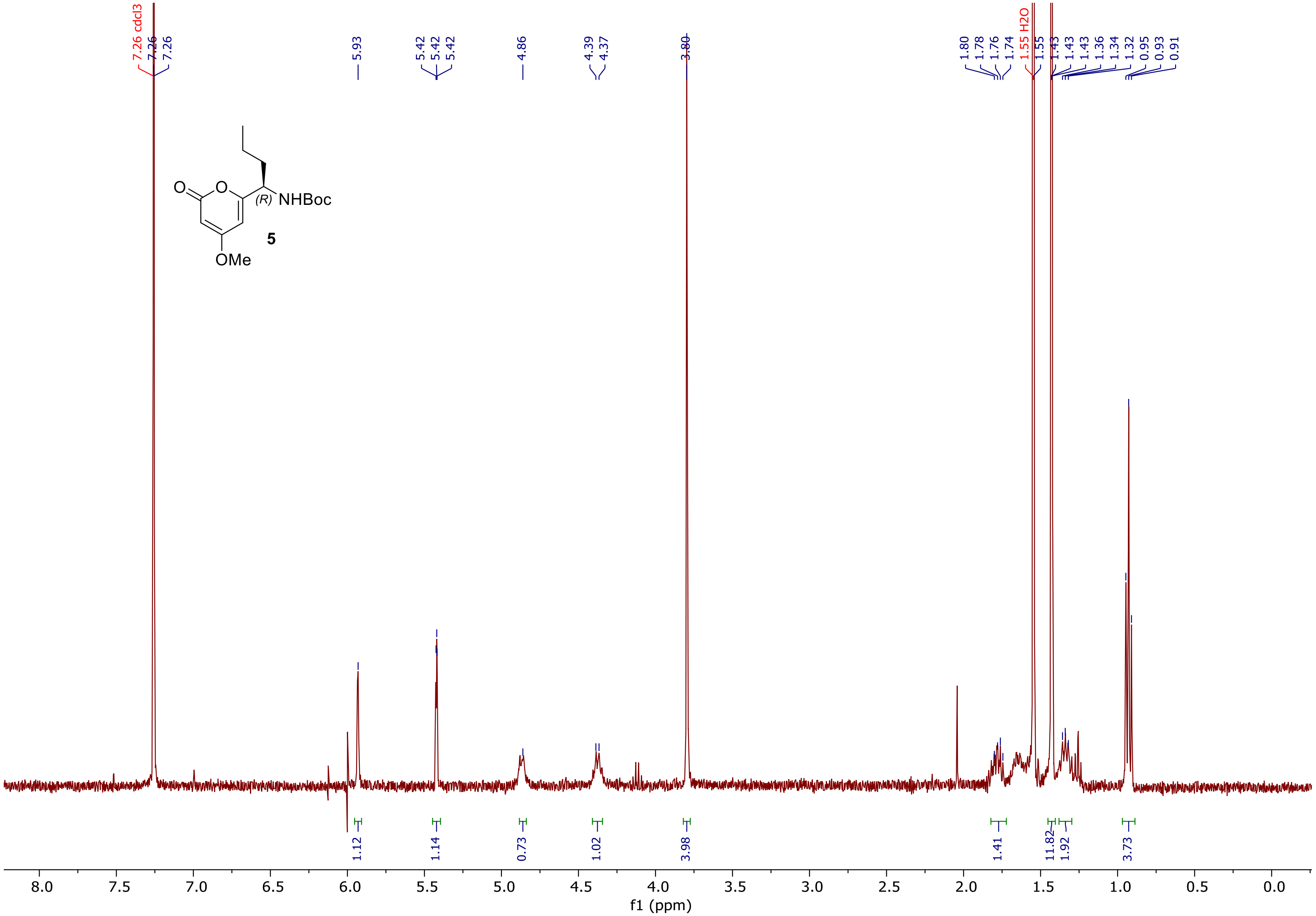

**Figure S7.** $^{13}$C NMR spectrum of *tert*-Butyl (*R*)-(1-(4-methoxy-2-oxo-2*H*-pyran-6-yl)butyl)carbamate (**5**). (75 MHz, $CDCl_3$).

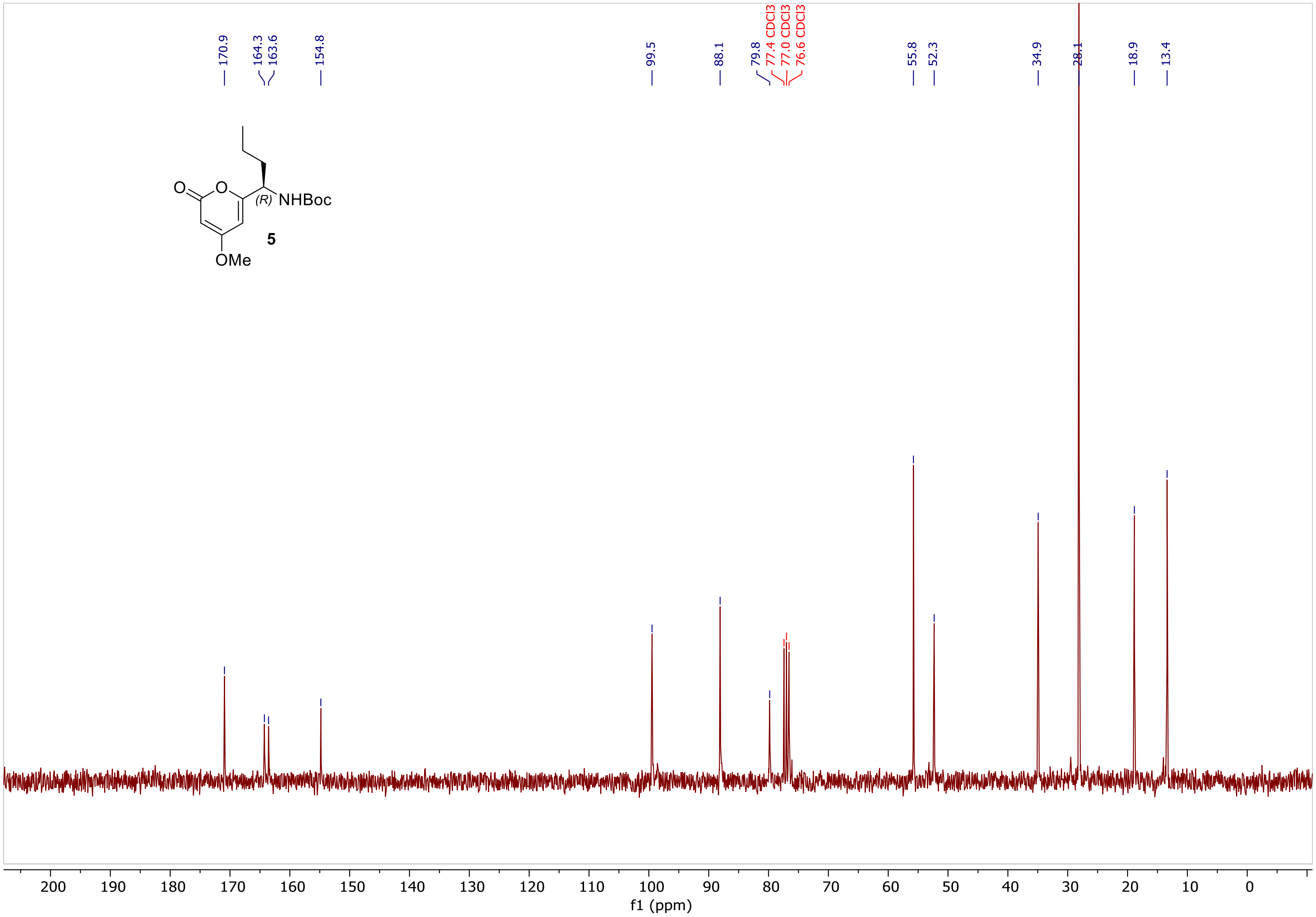

**Figure S8.** $^{1}$H NMR spectrum of (*R*)-1-(4-Methoxy-2-oxo-2*H*-pyran-6-yl)butan-1-aminium 2,2,2-trifluoroacetate (**6**). (300 MHz, $CDCl_3$).

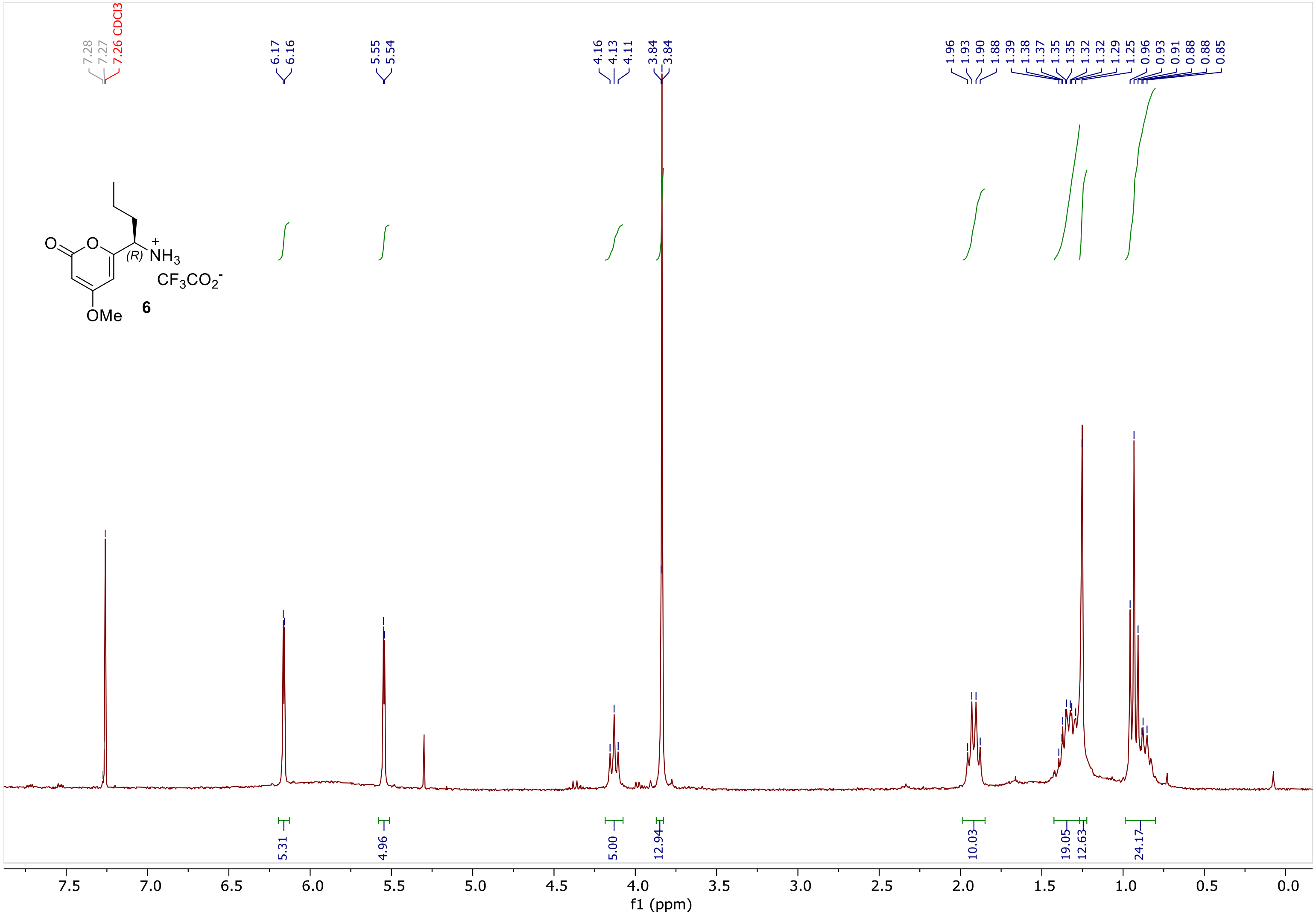

**Figure S9.** $^{13}$C NMR spectrum of (*R*)-1-(4-Methoxy-2-oxo-2*H*-pyran-6-yl)butan-1-aminium 2,2,2-trifluoroacetate (**6**). (75 MHz, $CDCl_3$).

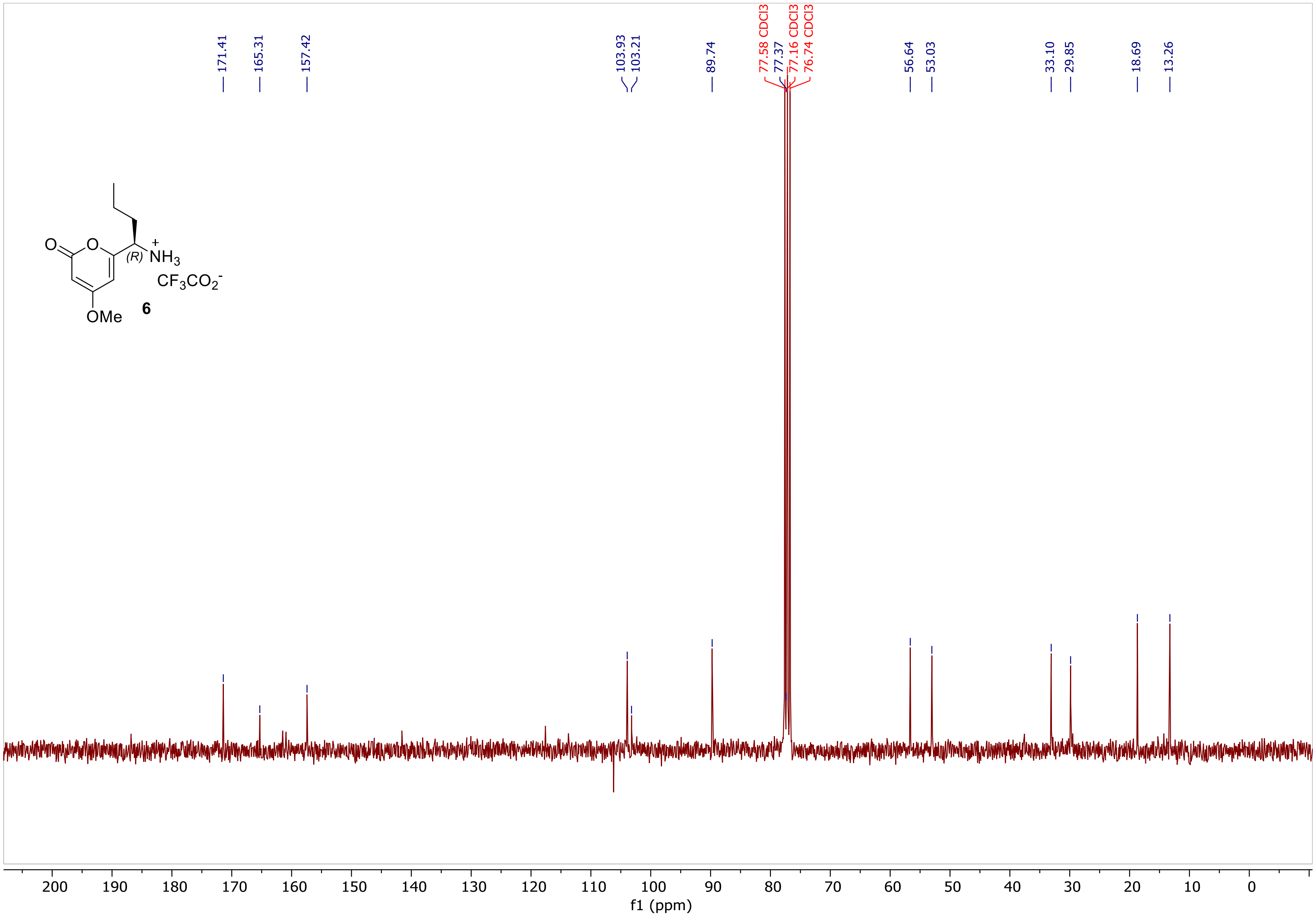

**Figure S10.** $^{1}$H NMR spectrum of (*R*)-2-(1,1-Diethoxyethyl)-4-methyl-4,5-dihydrothiazole-4-carboxylic acid (**7**). (300 MHz, $CDCl_3$).

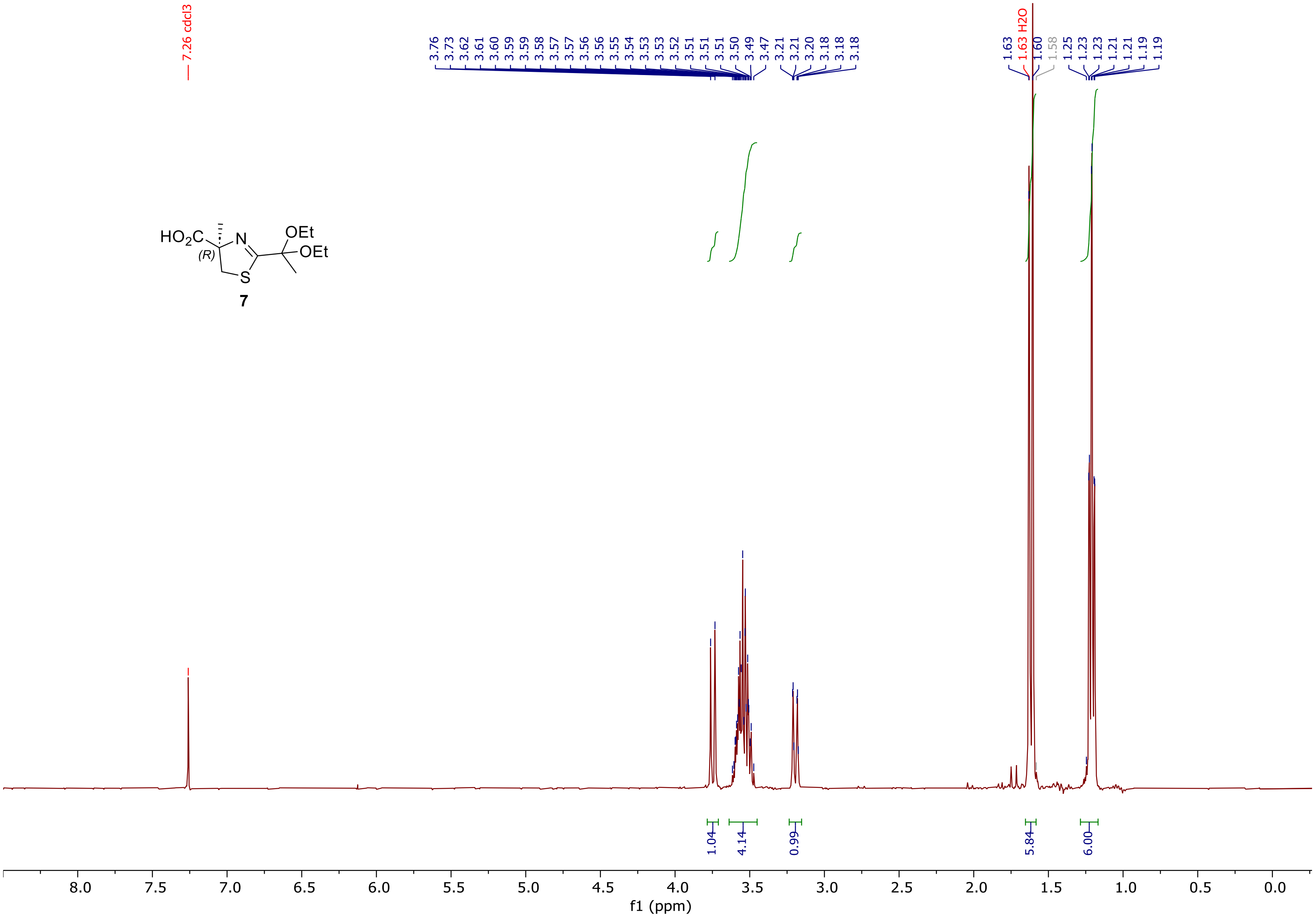

**Figure S11.** $^{13}$C NMR spectrum of (*R*)-2-(1,1-Diethoxyethyl)-4-methyl-4,5-dihydrothiazole-4-carboxylic acid (**7**). (75 MHz, $CDCl_3$).

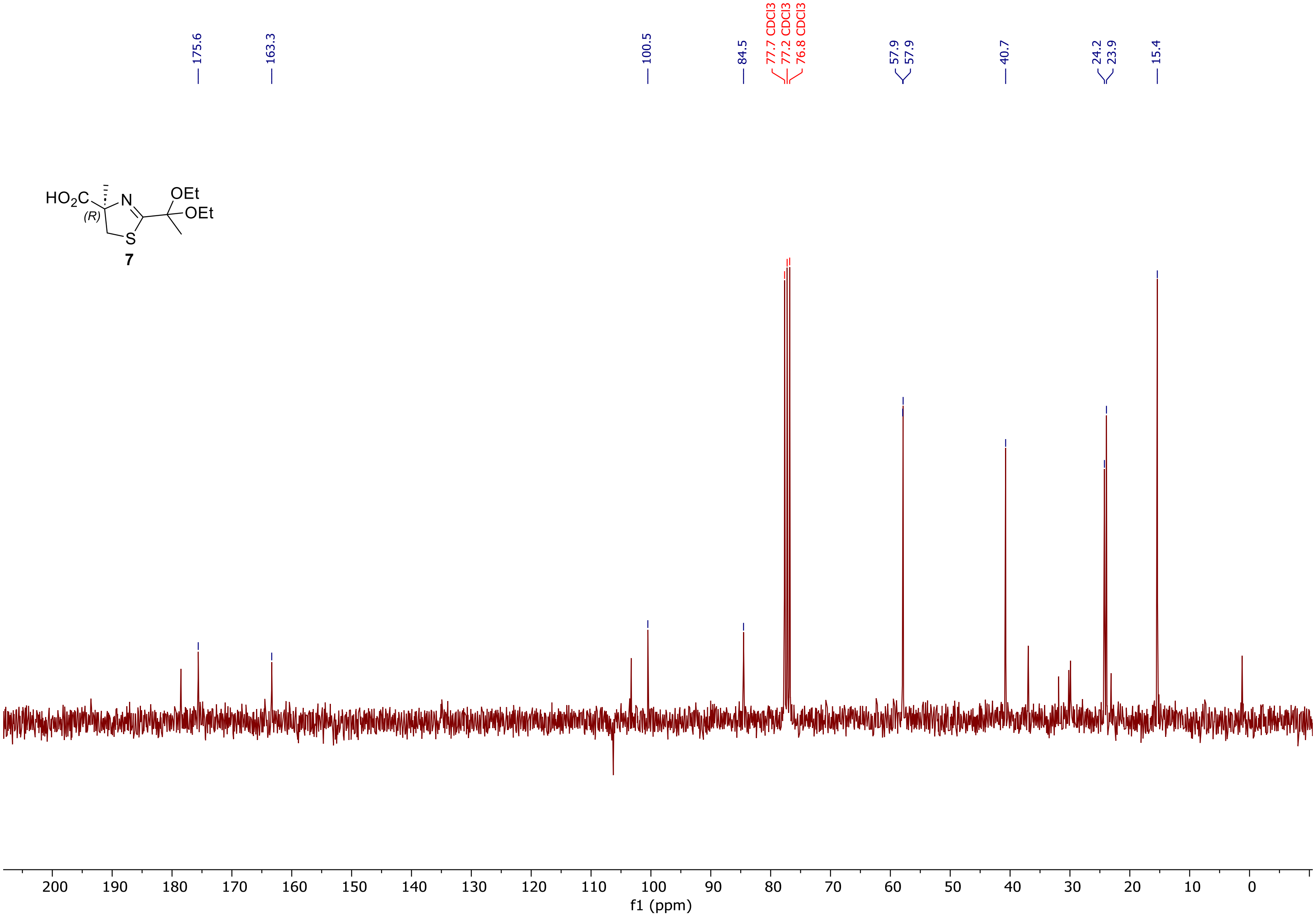

**Figure S12.** $^{1}$H NMR spectrum of (*R*)-2-(1,1-Diethoxyethyl)-*N*-((*R*)-1-(4-methoxy-2-oxo-2*H*-pyran-6-yl)butyl)-4-methyl-4,5-dihydrothiazole-4-carboxamide (**8**). (400 MHz, $CDCl_3$).

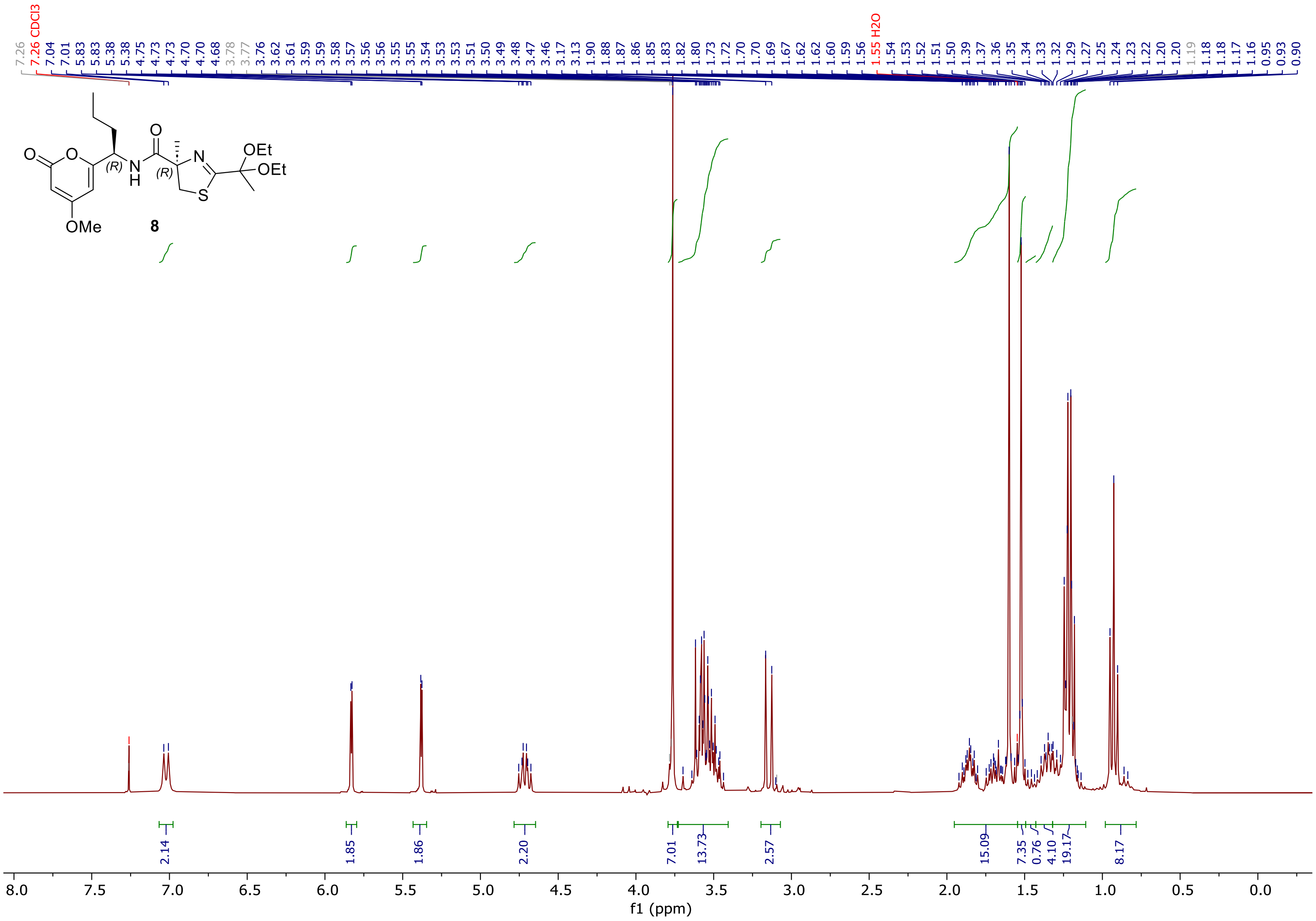

**Figure S13.** $^{13}$C NMR spectrum of (*R*)-2-(1,1-Diethoxyethyl)-*N*-((*R*)-1-(4-methoxy-2-oxo-2*H*-pyran-6-yl)butyl)-4-methyl-4,5-dihydrothiazole-4-carboxamide (**8**). (75 MHz, $CDCl_3$).

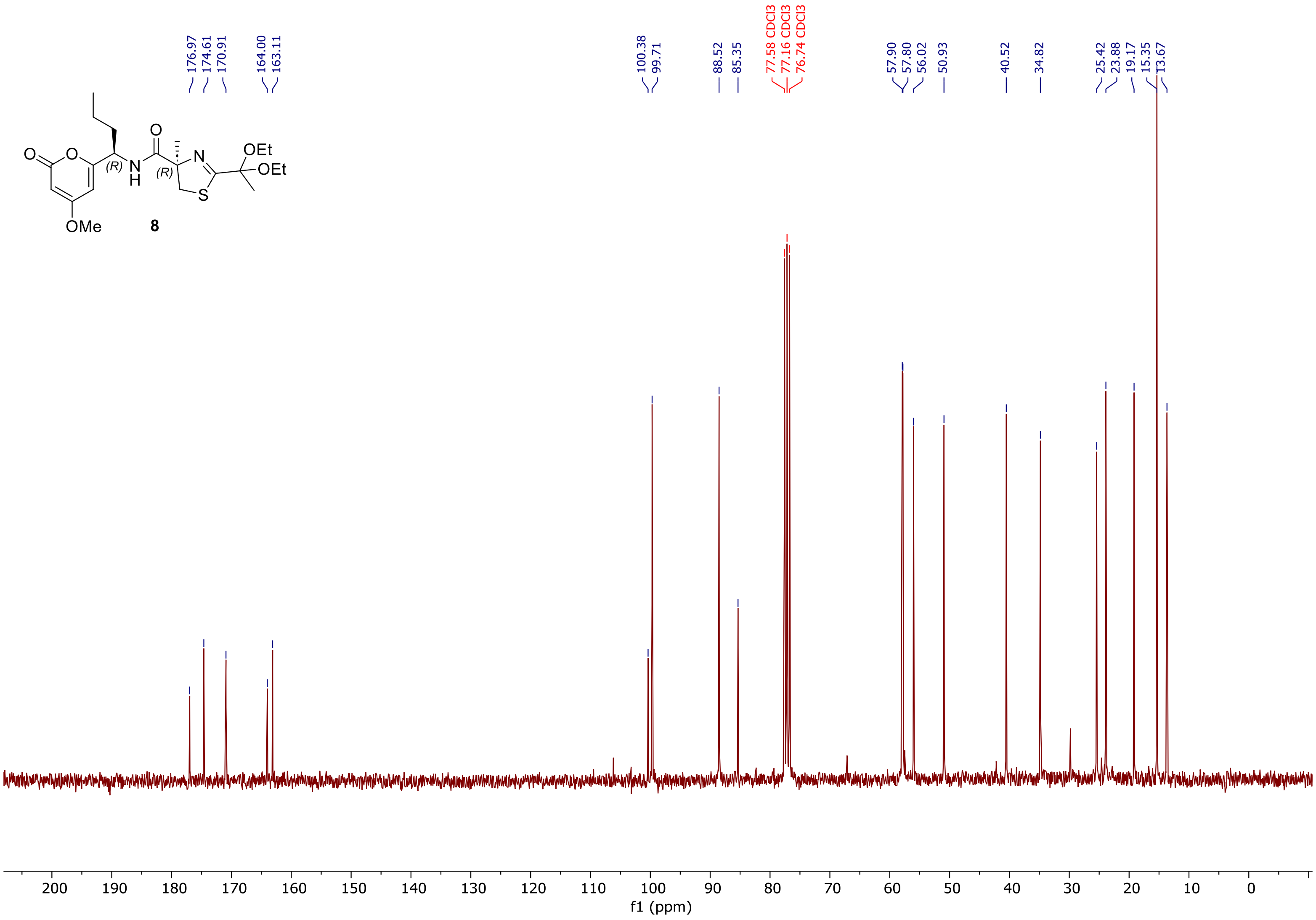

**Figure S14.** $^{1}$H NMR spectrum of (*R*)-2-Acetyl-*N*-((*R*)-1-(4-methoxy-2-oxo-2*H*-pyran-6-yl)butyl)-4-methyl-4,5-dihydrothiazole-4-carboxamide (**9**). (400 MHz, $CDCl_3$).

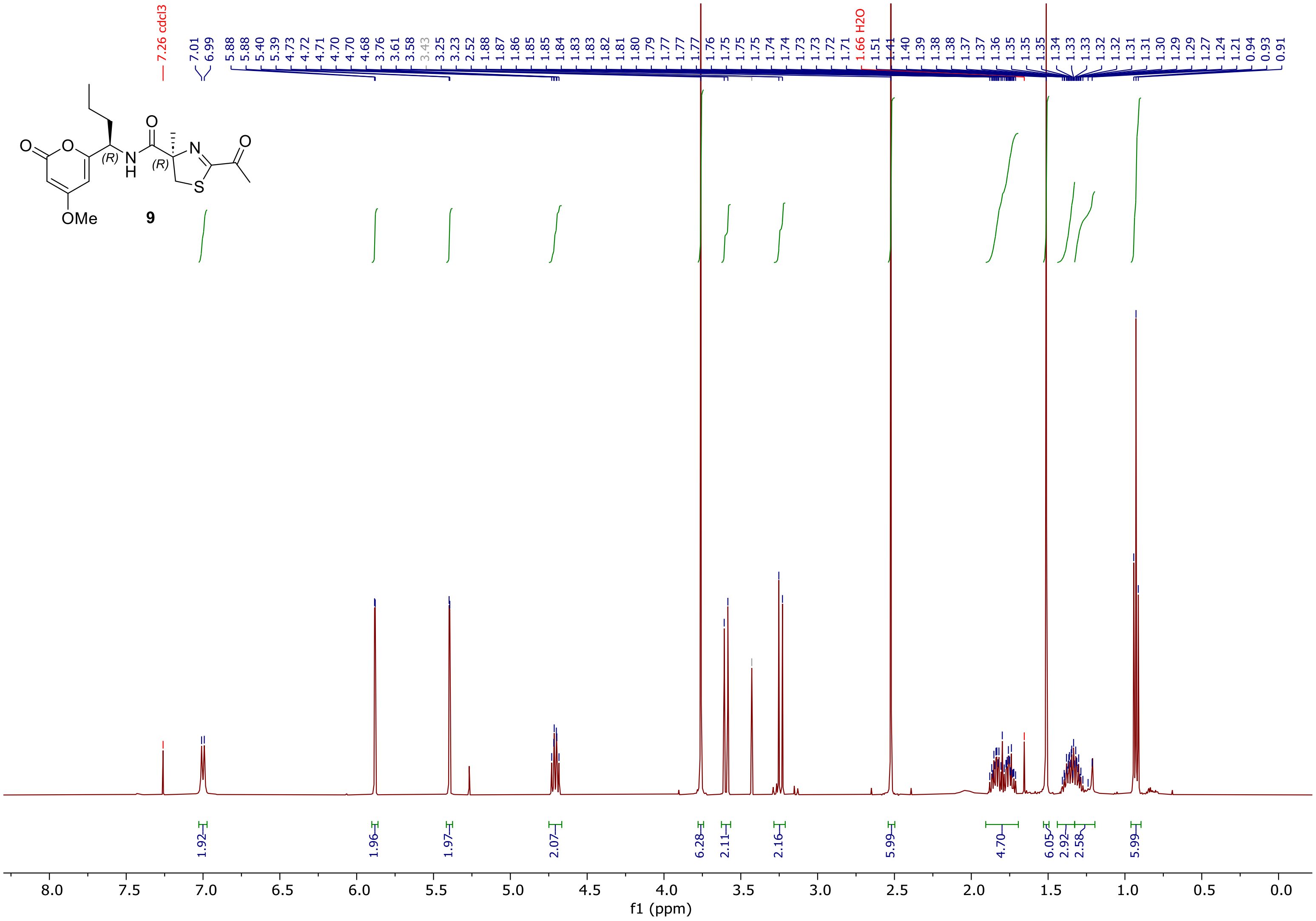

**Figure S15.** $^{13}$C NMR spectrum of (*R*)-2-Acetyl-*N*-((*R*)-1-(4-methoxy-2-oxo-2*H*-pyran-6-yl)butyl)-4-methyl-4,5-dihydrothiazole-4-carboxamide (**9**). (75 MHz, $CDCl_3$).

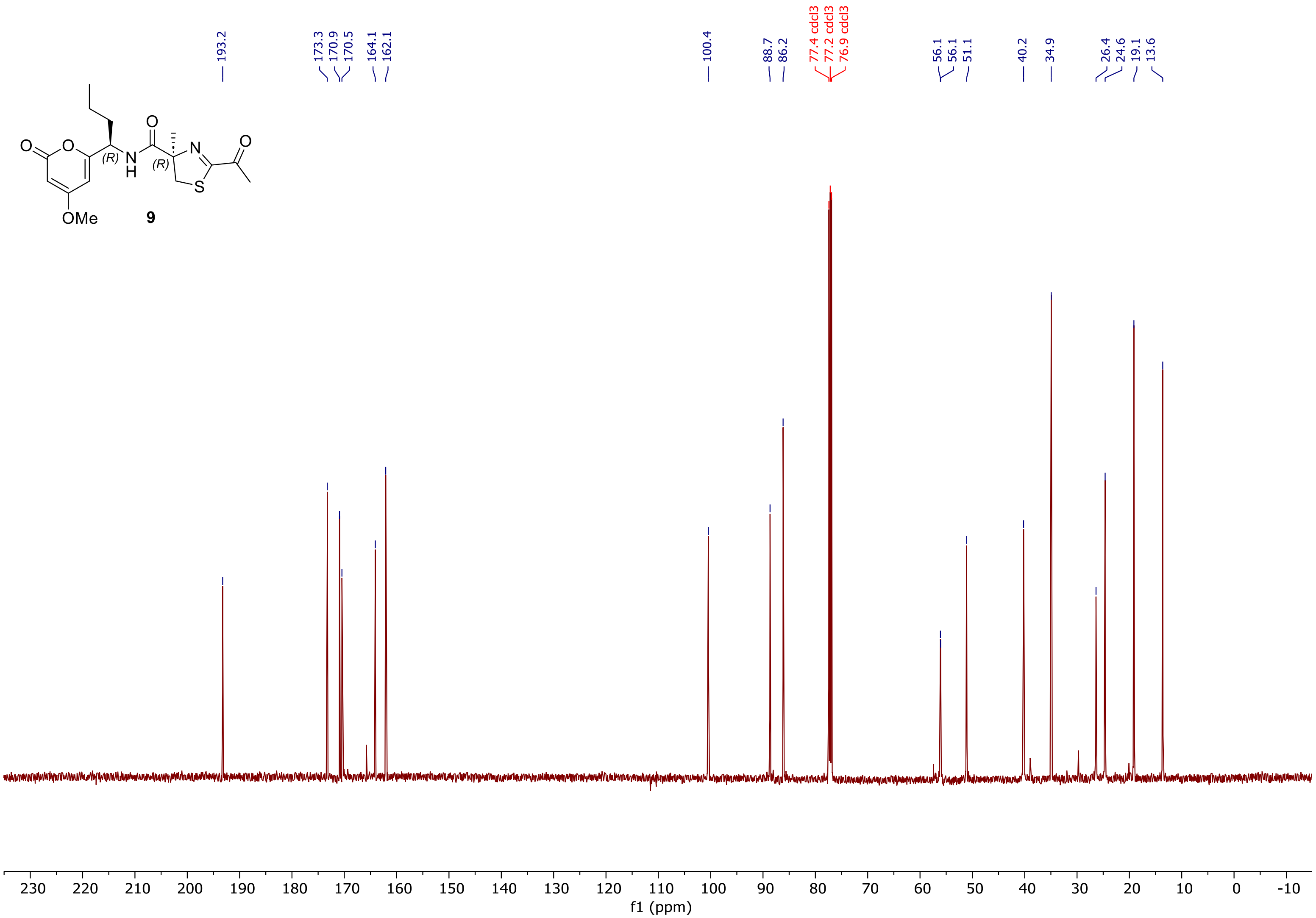

**Figure S16.** $^{1}$H NMR spectrum of (*R*)-2-((*E*)-1-(Hydroxyimino)ethyl)-*N*-((*R*)-1-(4-methoxy-2-oxo-2*H*-pyran-6-yl)butyl)-4-methyl-4,5-dihydrothiazole-4-carboxamide (**PM742**). (500 MHz, $CD_3OD$).

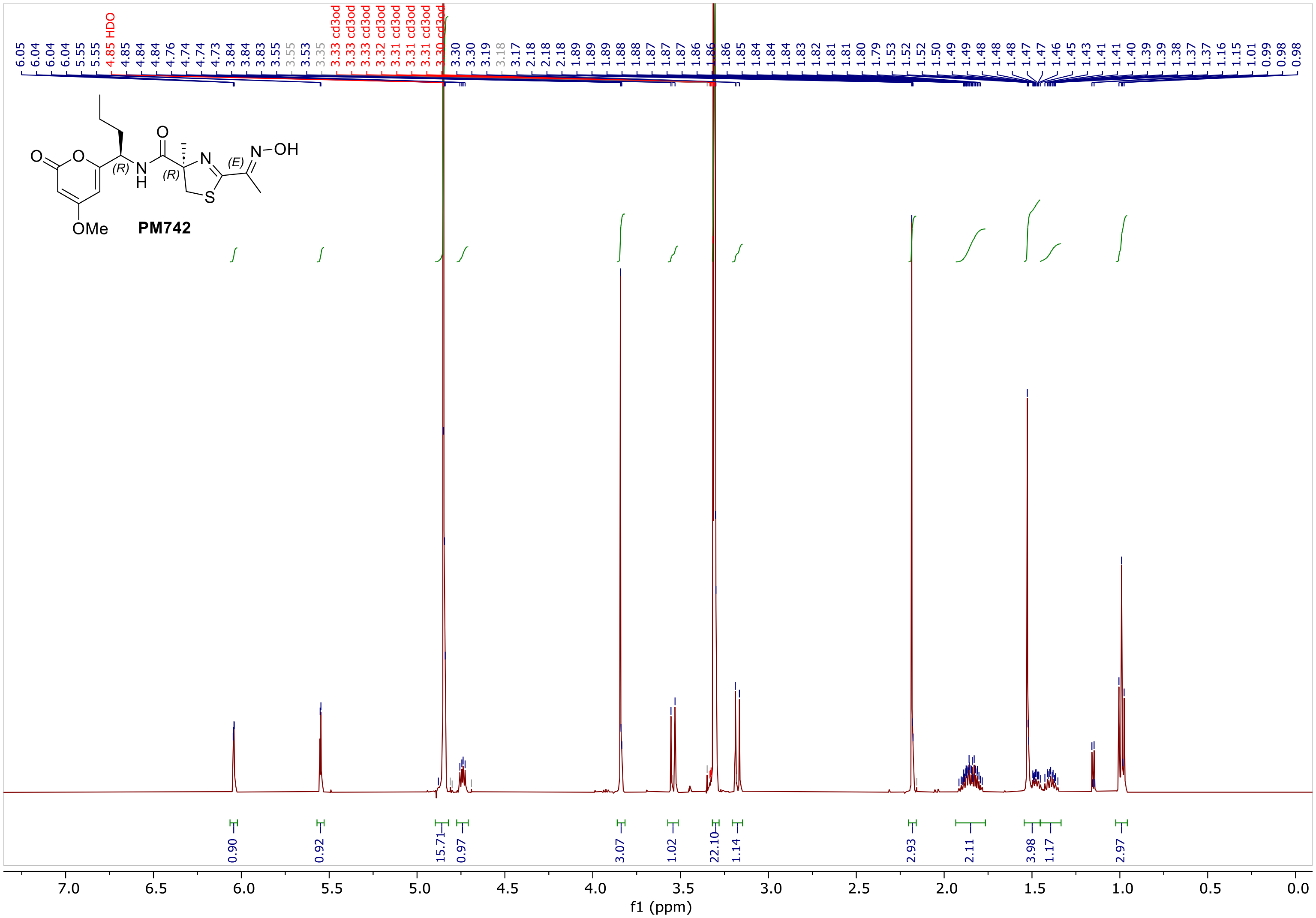

**Figure S17.** $^{13}$C NMR spectrum of (*R*)-2-((*E*)-1-(Hydroxyimino)ethyl)-*N*-((*R*)-1-(4-methoxy-2-oxo-2*H*-pyran-6-yl)butyl)-4-methyl-4,5-dihydrothiazole-4-carboxamide (**PM742**). (125 MHz, $CD_3OD$).

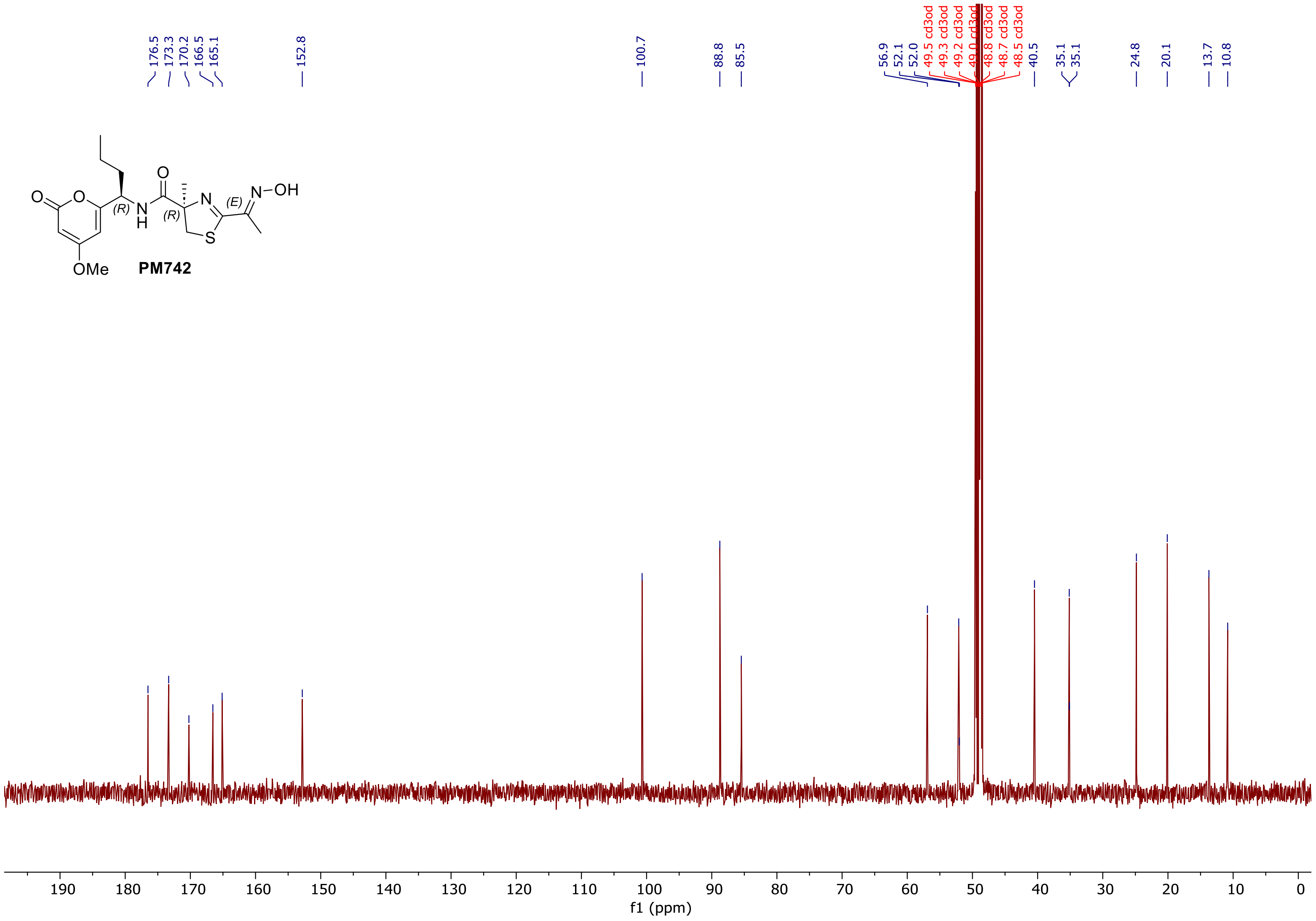

**Figure S18.** $^{1}$H NMR spectrum of (*R*)-2-((*Z*)-1-(Hydroxyimino)ethyl)-*N*-((*R*)-1-(4-methoxy-2-oxo-2*H*-pyran-6-yl)butyl)-4-methyl-4,5-dihydrothiazole-4-carboxamide (*Z*-**PM742**). (500 MHz, $CD_3OD$).

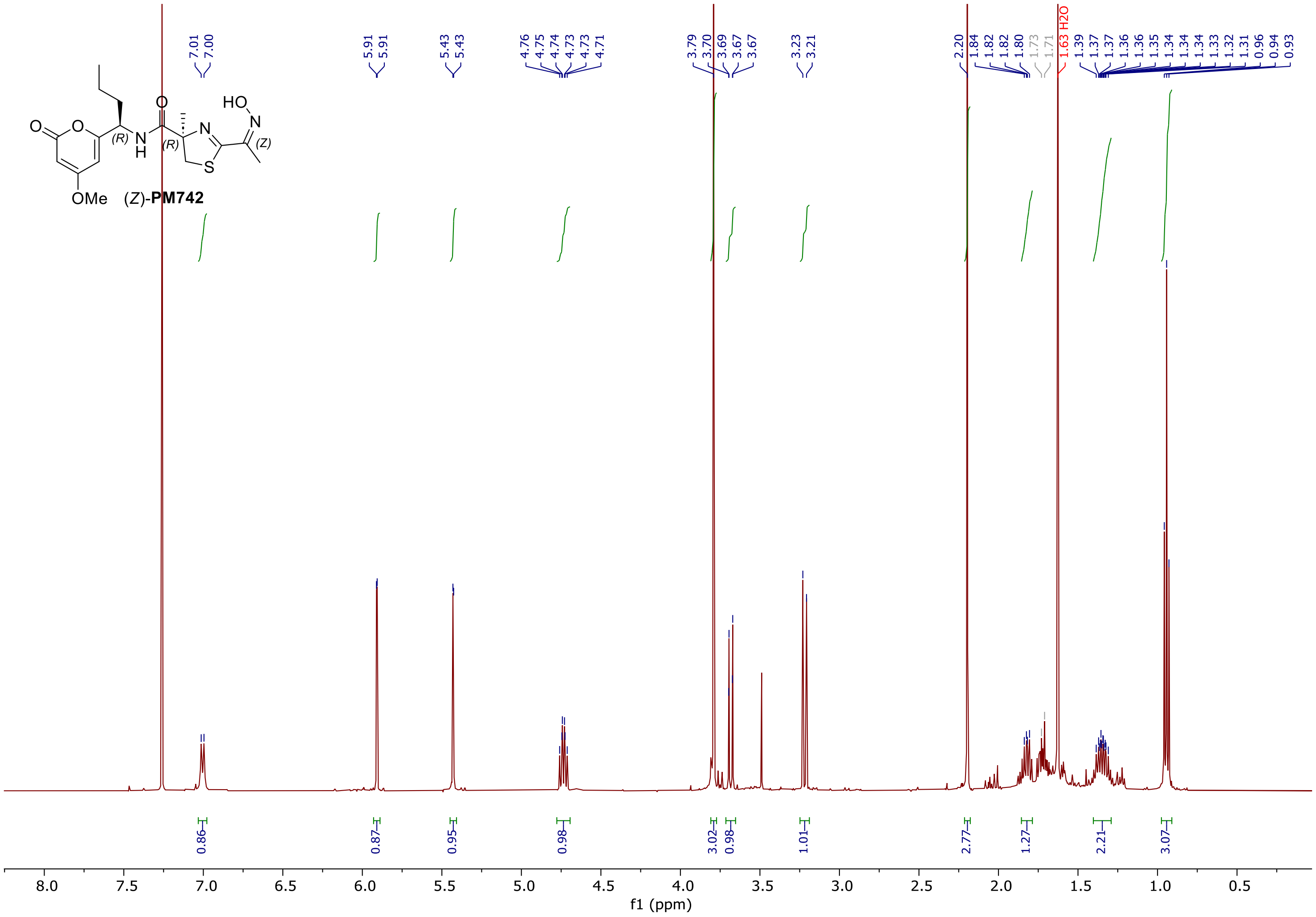

**Figure S19.** $^{13}$C NMR spectrum of (*R*)-2-((*Z*)-1-(Hydroxyimino)ethyl)-*N*-((*R*)-1-(4-methoxy-2-oxo-2*H*-pyran-6-yl)butyl)-4-methyl-4,5-dihydrothiazole-4-carboxamide (*Z*-**PM742**). (75 MHz, $CDCl_3$).

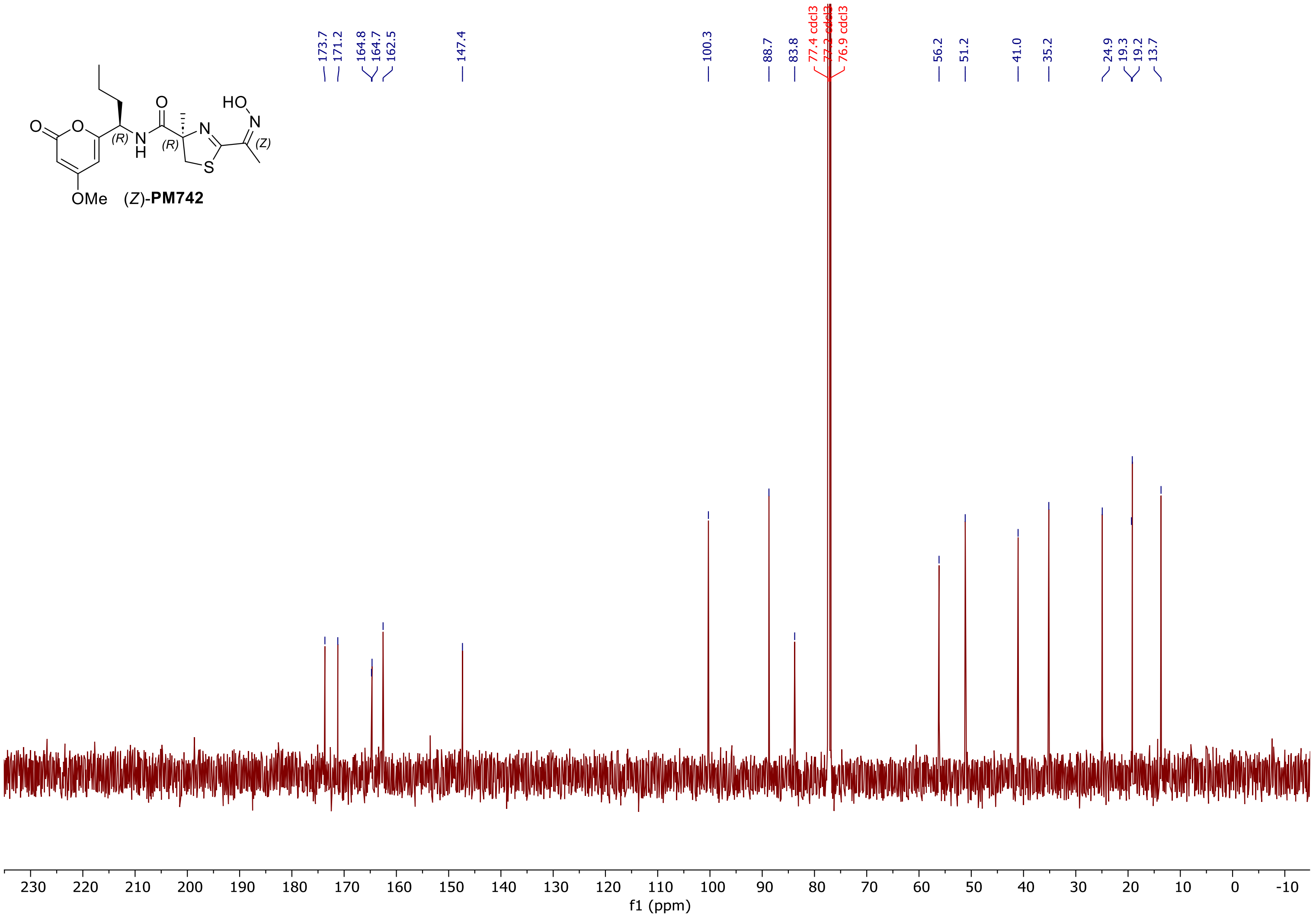

**Figure S20.** ROESY spectrum of (*R*)-2-((*Z*)-1-(Hydroxyimino)ethyl)-*N*-((*R*)-1-(4-methoxy-2-oxo-2*H*-pyran-6-yl)butyl)-4-methyl-4,5-dihydrothiazole-4-carboxamide (*Z*-**PM742**). (500 MHz, $(CD_3)_2SO$).

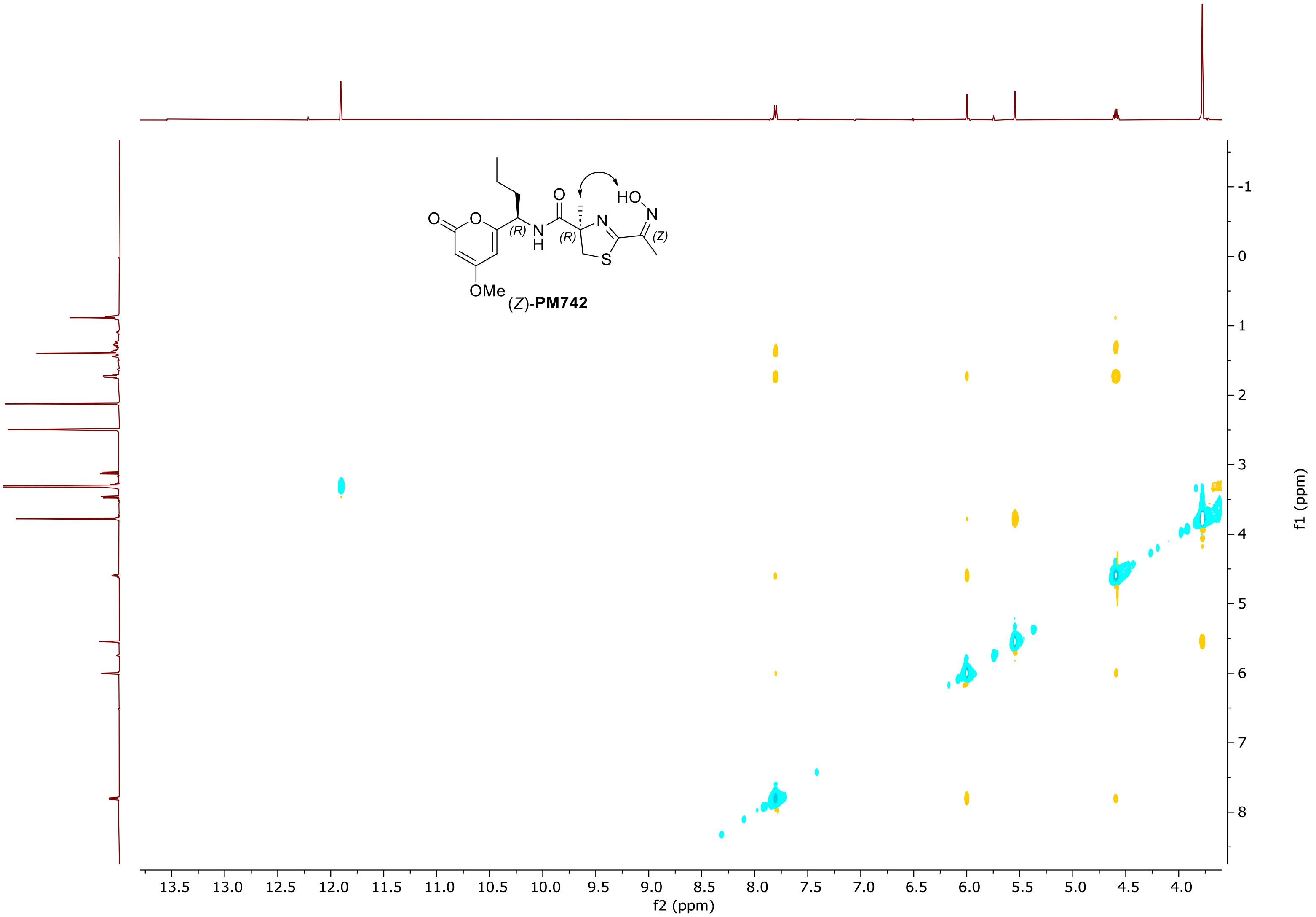

**Figure S21.** $^{1}$H NMR spectrum of *tert*-Butyl (*R*)-(1-(4-(cyclopropylmethoxy)-2-oxo-2*H*-pyran-6-yl)butyl)carbamate (**10**). (400 MHz, $CDCl_3$).

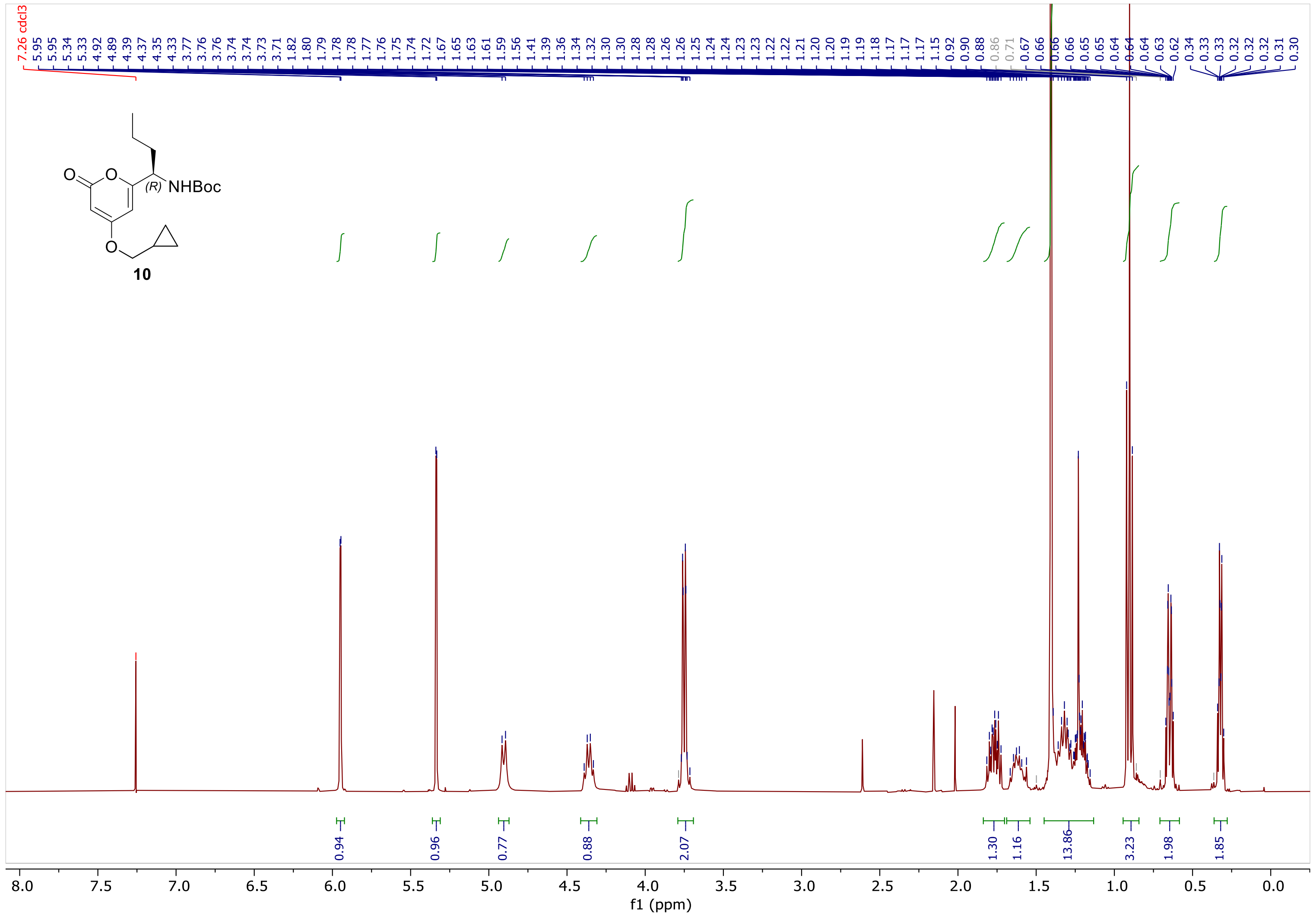

**Figure S22.** $^{13}C$ NMR spectrum of *tert*-Butyl (*R*)-(1-(4-(cyclopropylmethoxy)-2-oxo-2*H*-pyran-6-yl)butyl)carbamate (**10**). (100 MHz, $CDCl_3$).

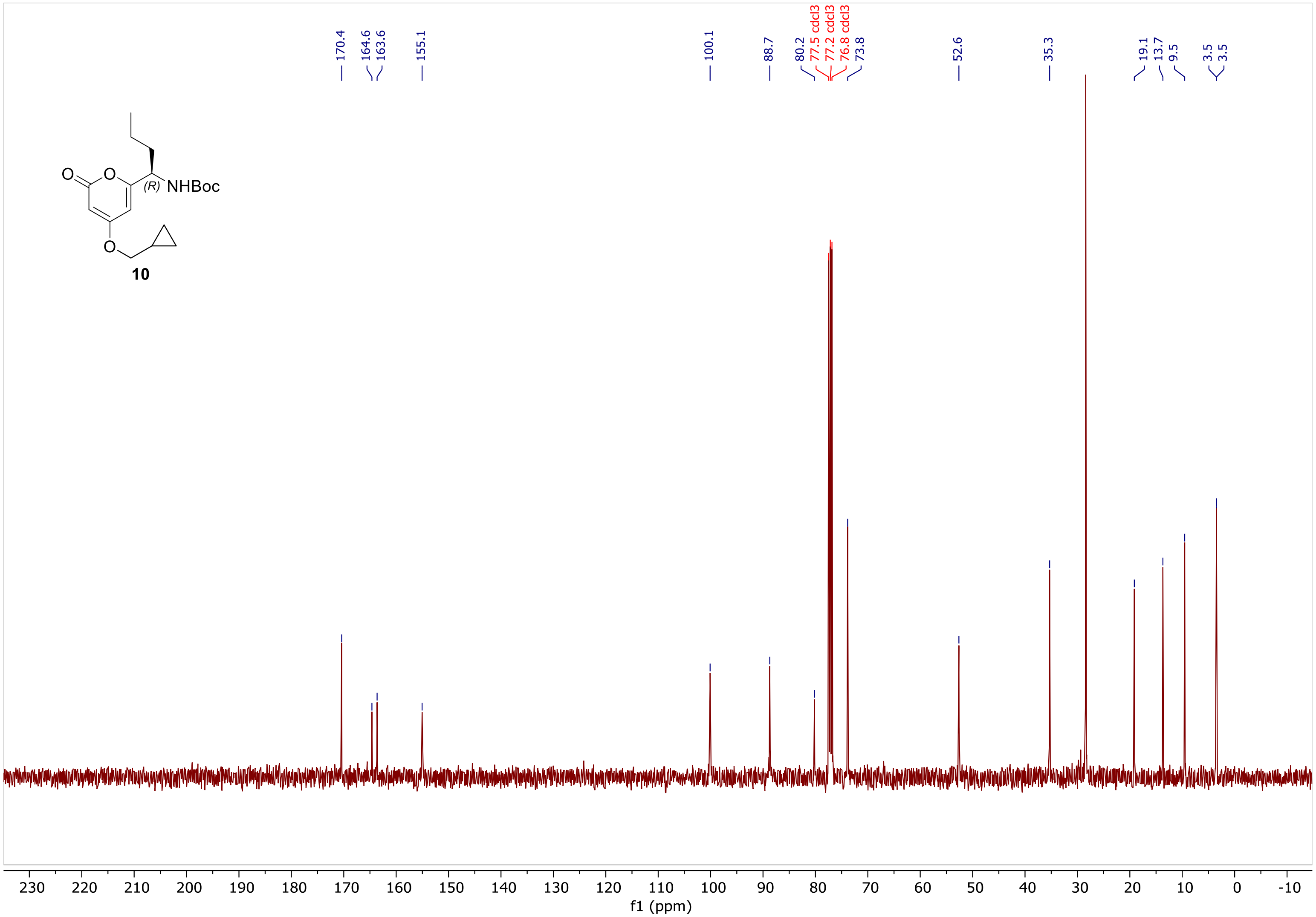

**Figure S23.** $^1$H NMR spectrum of (*R*)-1-(4-(Cyclopropylmethoxy)-2-oxo-2*H*-pyran-6-yl)butan-1-aminium 2,2,2-trifluoroacetate (**11**). (300 MHz, $CDCl_3$).

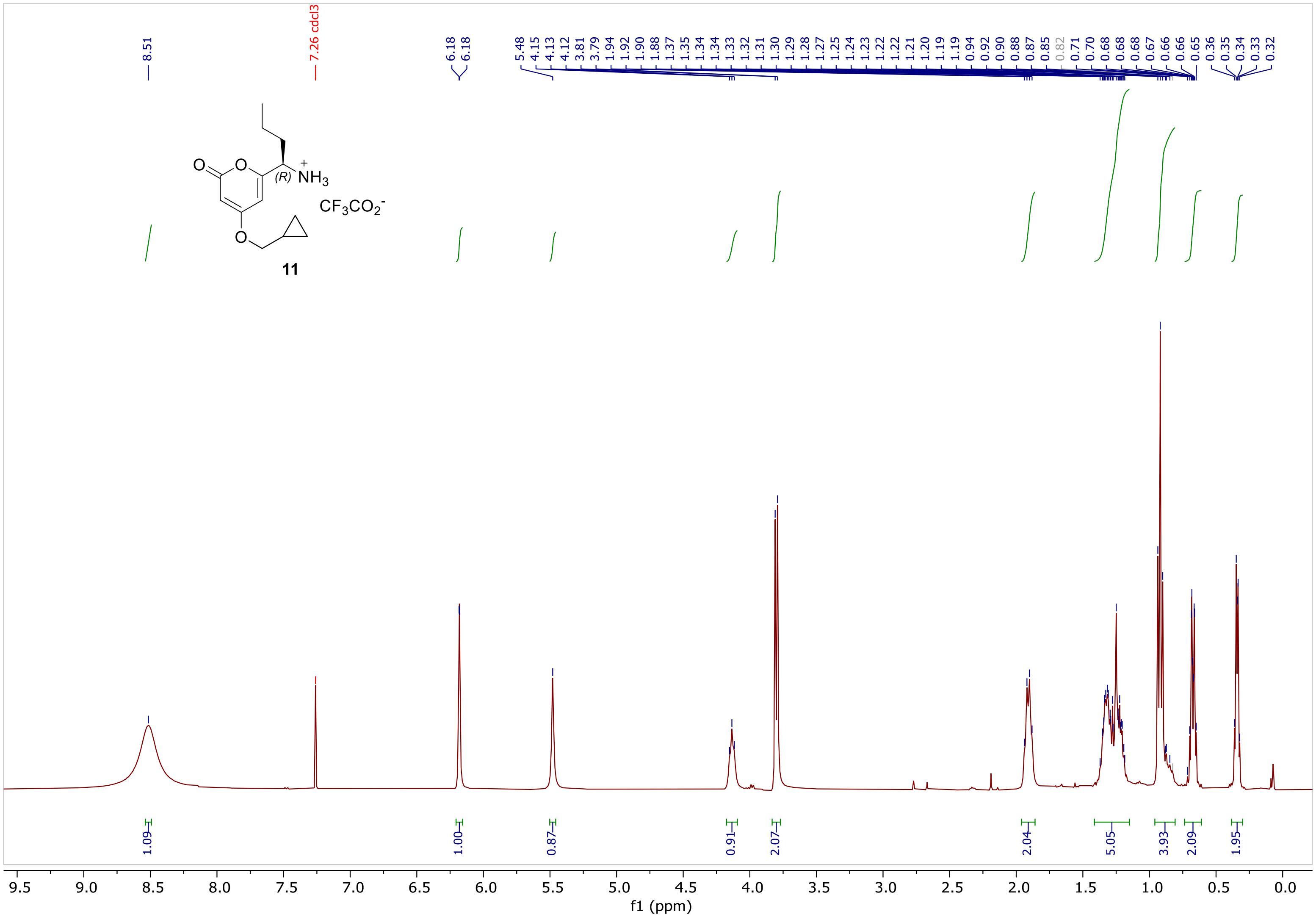

**Figure S24.** $^{13}$C NMR spectrum of (*R*)-1-(4-(Cyclopropylmethoxy)-2-oxo-2*H*-pyran-6-yl)butan-1-aminium 2,2,2-trifluoroacetate (**11**). (75 MHz, $CDCl_3$).

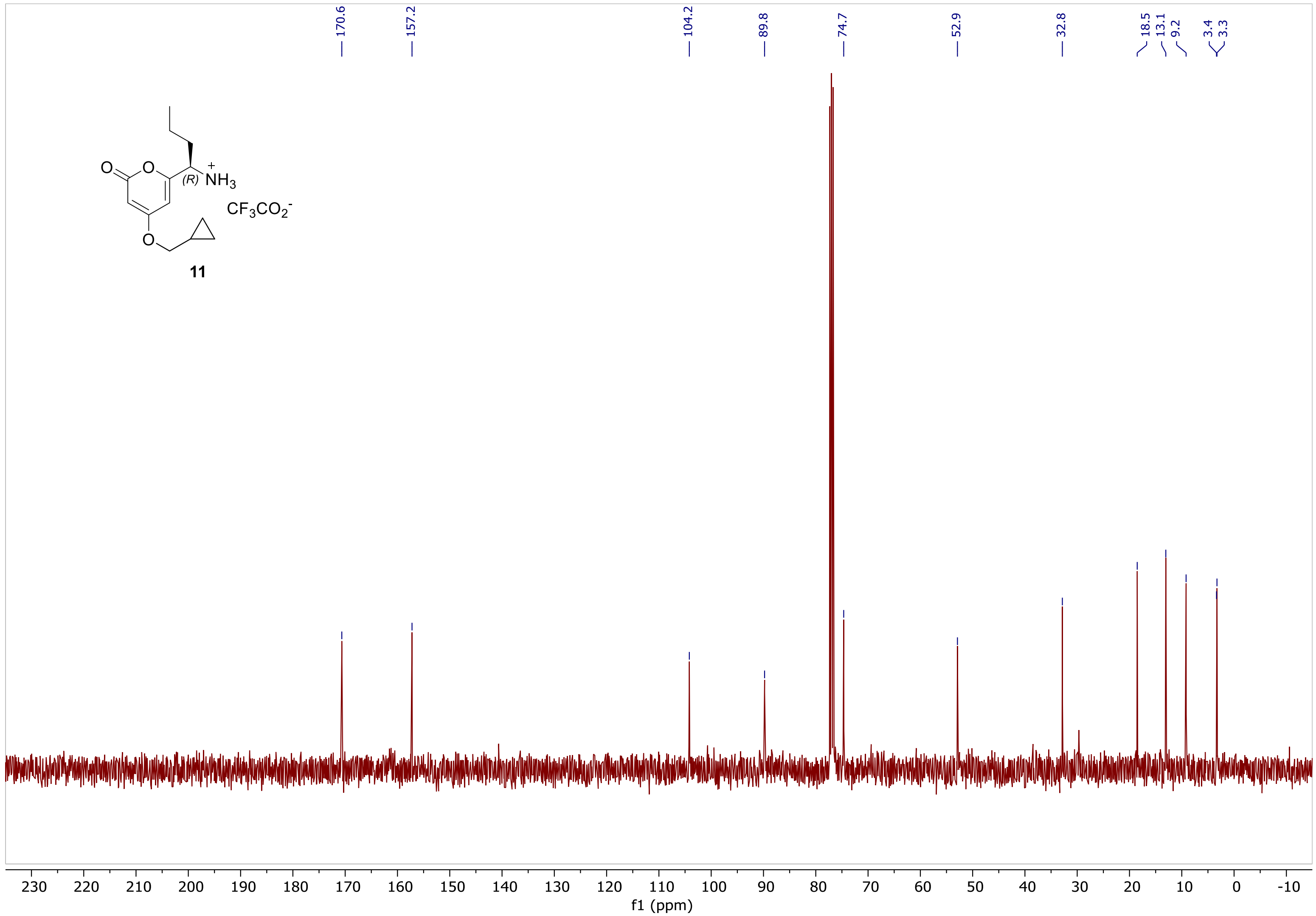

**Figure S25.** $^{1}$H NMR spectrum of (*R*)-*N*-((*R*)-1-(4-(Cyclopropylmethoxy)-2-oxo-2*H*-pyran-6-yl)butyl)-2-(1,1-diethoxyethyl)-4-methyl-4,5-dihydrothiazole-4-carboxamide (**12**). (400 MHz, $CDCl_3$).

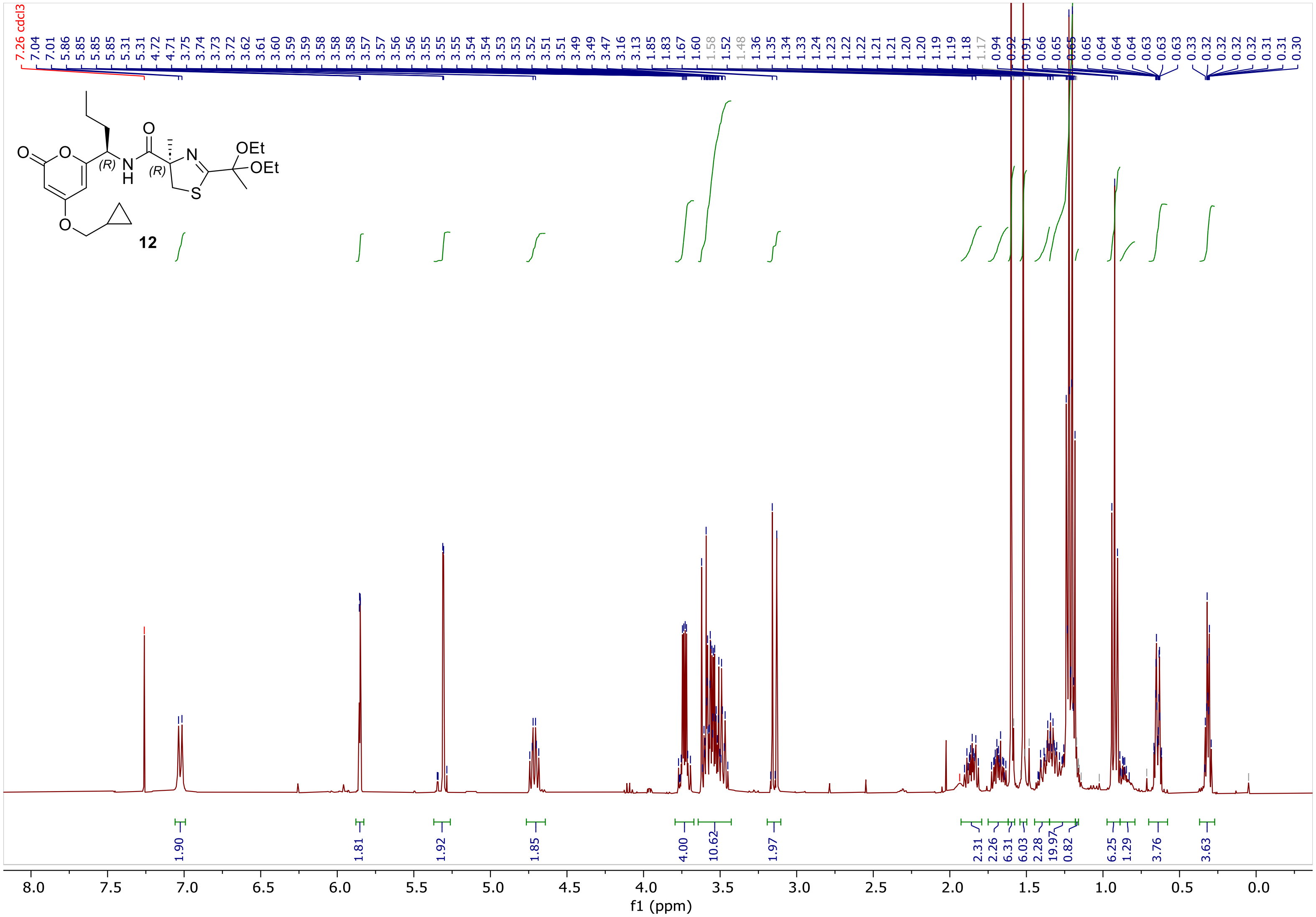

**Figure S26.** $^{13}$C NMR spectrum of (*R*)-*N*-((*R*)-1-(4-(Cyclopropylmethoxy)-2-oxo-2*H*-pyran-6-yl)butyl)-2-(1,1-diethoxyethyl)-4-methyl-4,5-dihydrothiazole-4-carboxamide (**12**). (100 MHz, CDCl$_3$).

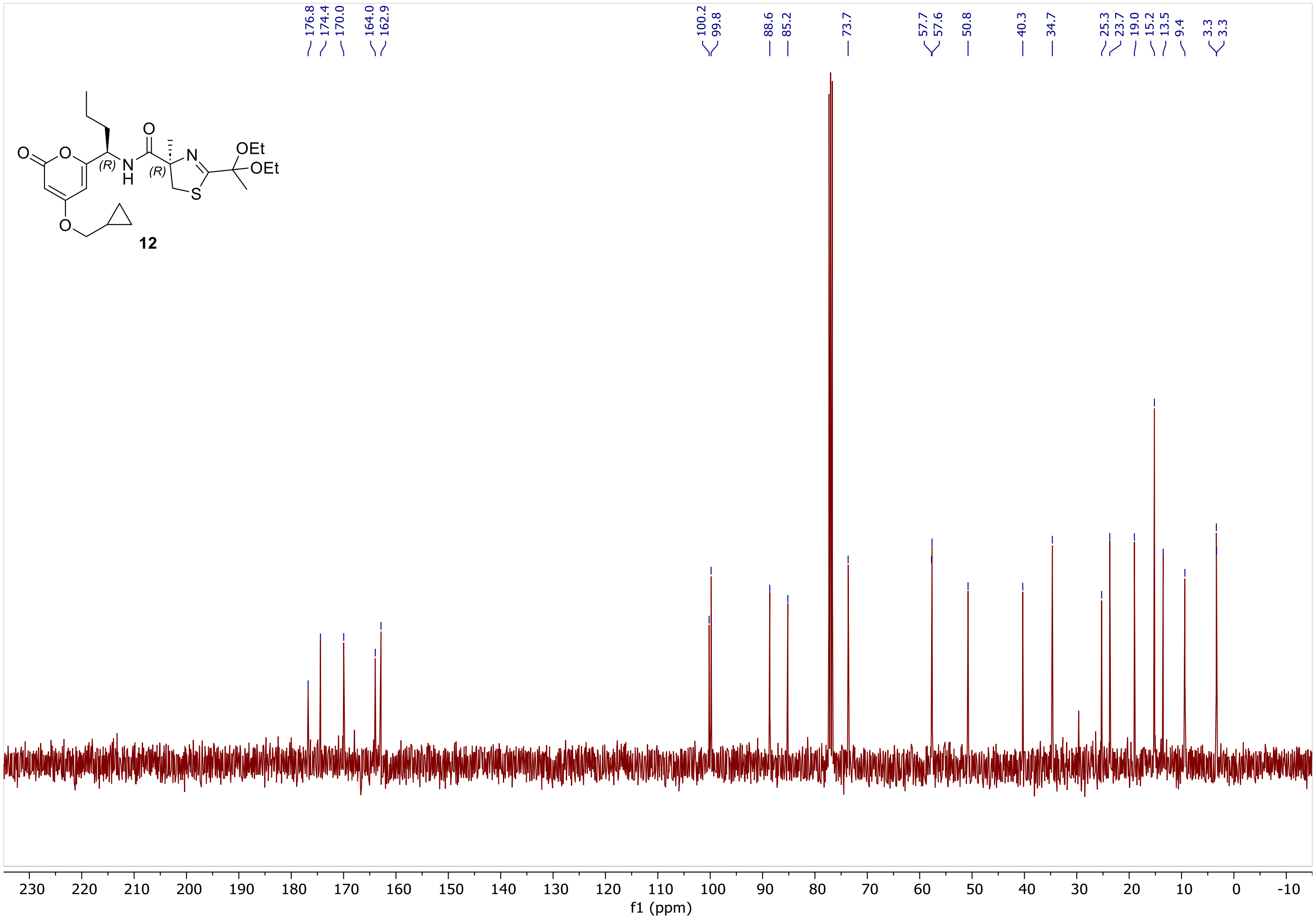

**Figure S27.** $^1$H NMR spectrum of (*R*)-2-Acetyl-*N*-((*R*)-1-(4-(cyclopropylmethoxy)-2-oxo-2*H*-pyran-6-yl)butyl)-4-methyl-4,5-dihydrothiazole-4-carboxamide (**13**). (400 MHz, $CDCl_3$).

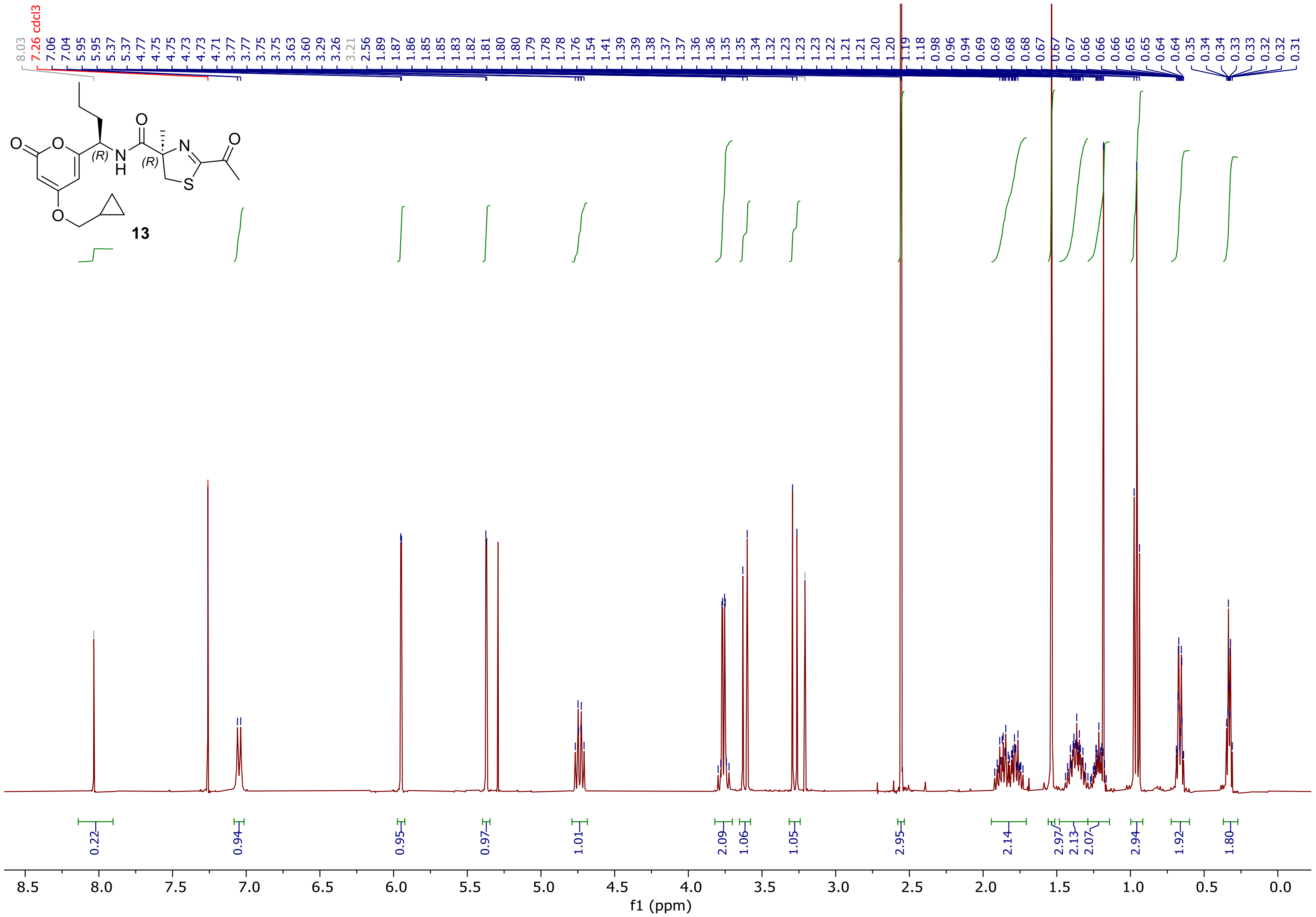

**Figure S28.** $^{13}$C NMR spectrum of (*R*)-2-Acetyl-*N*-((*R*)-1-(4-(cyclopropylmethoxy)-2-oxo-2*H*-pyran-6-yl)butyl)-4-methyl-4,5-dihydrothiazole-4-carboxamide (**13**). (100 MHz, $CDCl_3$).

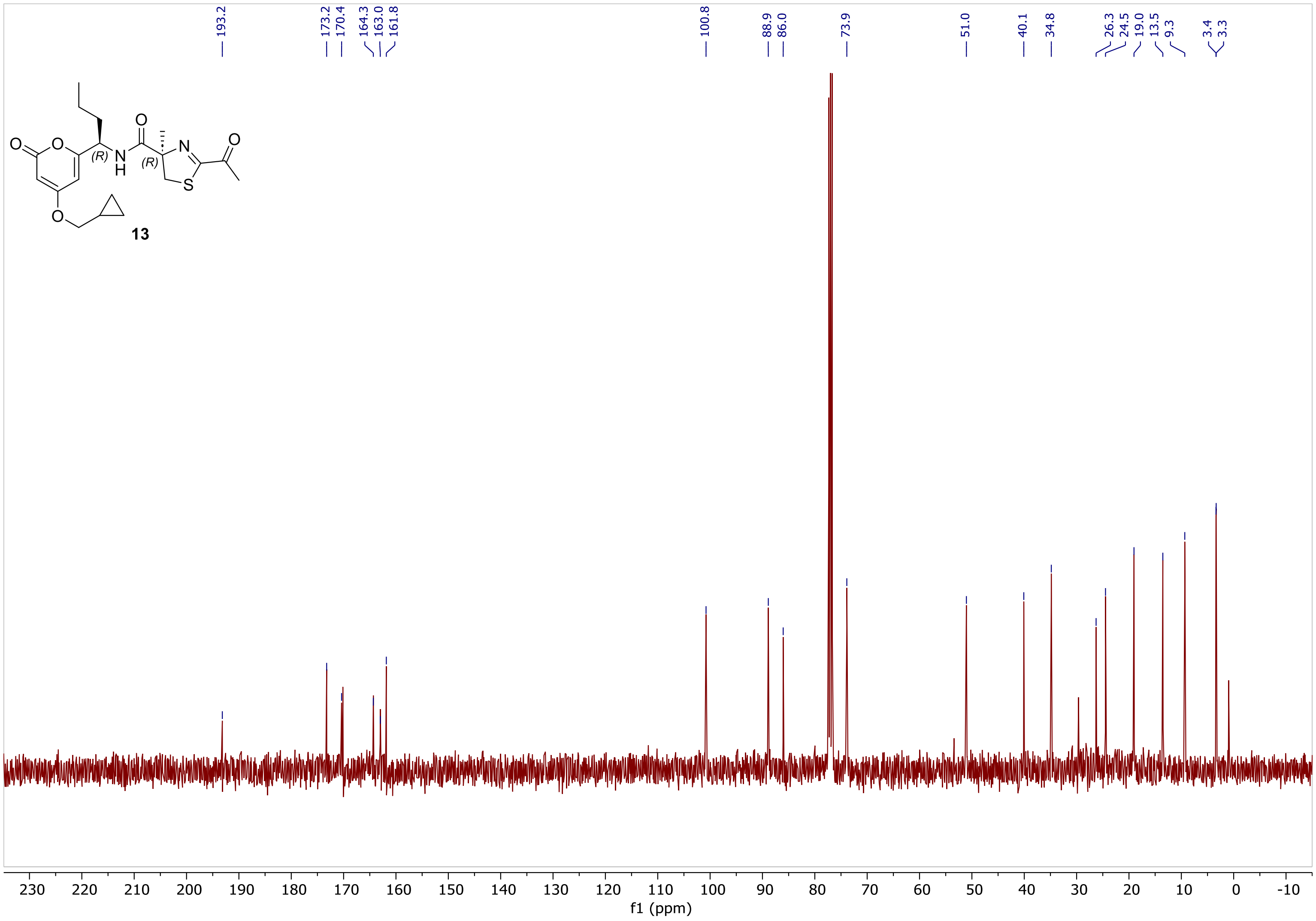

**Figure S29.** $^{1}$H NMR spectrum of (*R*)-*N*-((*R*)-1-(4-(Cyclopropylmethoxy)-2-oxo-2*H*-pyran-6-yl)butyl)-2-((*E*)-1-(hydroxyimino)ethyl)-4-methyl-4,5-dihydrothiazole-4-carboxamide (**PM534**). (400 MHz, $CD_3OD$).

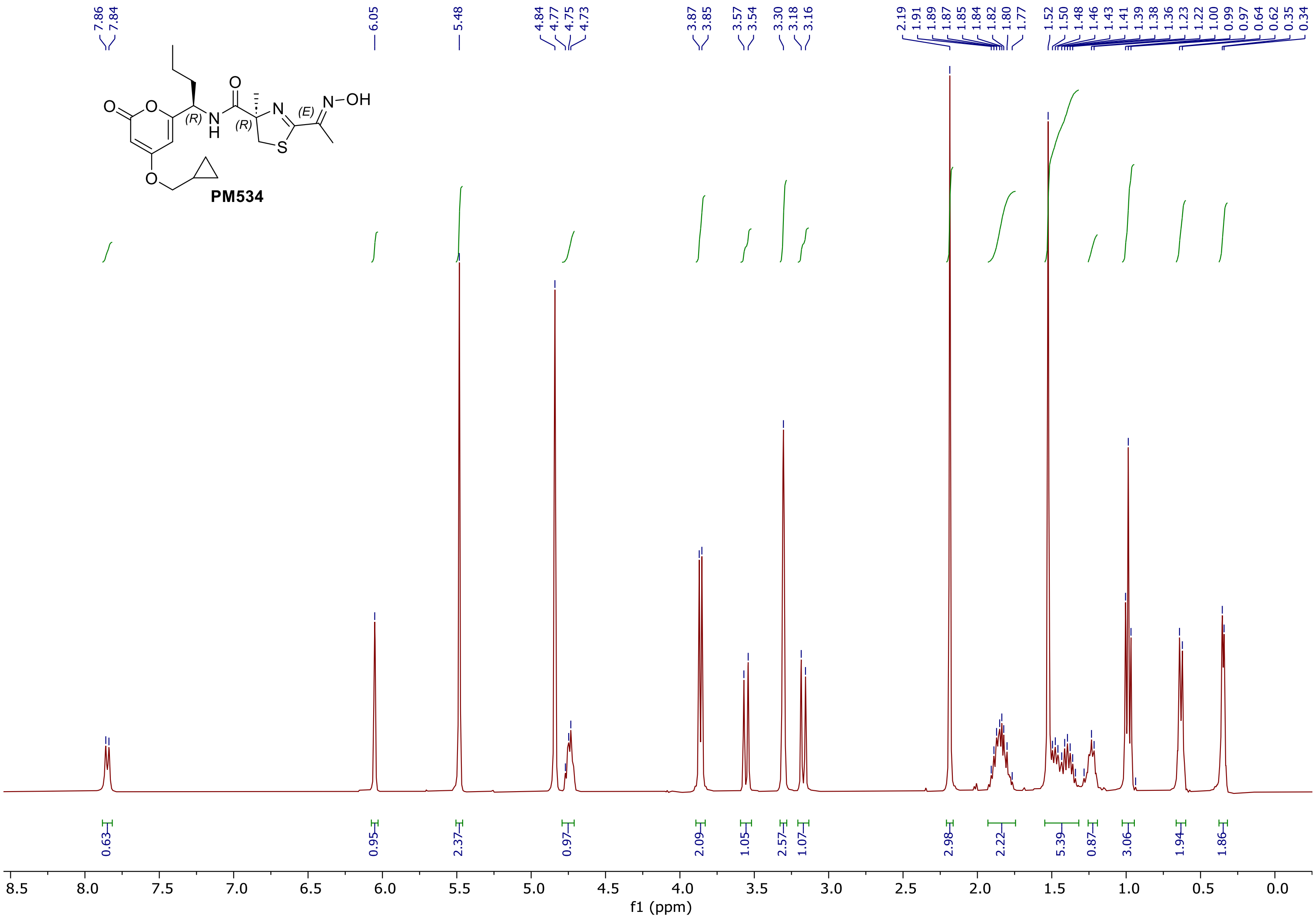

**Figure S30.** $^{13}$C NMR spectrum of (*R*)-*N*-((*R*)-1-(4-(Cyclopropylmethoxy)-2-oxo-2*H*-pyran-6-yl)butyl)-2-((*E*)-1-(hydroxyimino)ethyl)-4-methyl-4,5-dihydrothiazole-4-carboxamide (**PM534**). (100 MHz, $CDCl_3$).

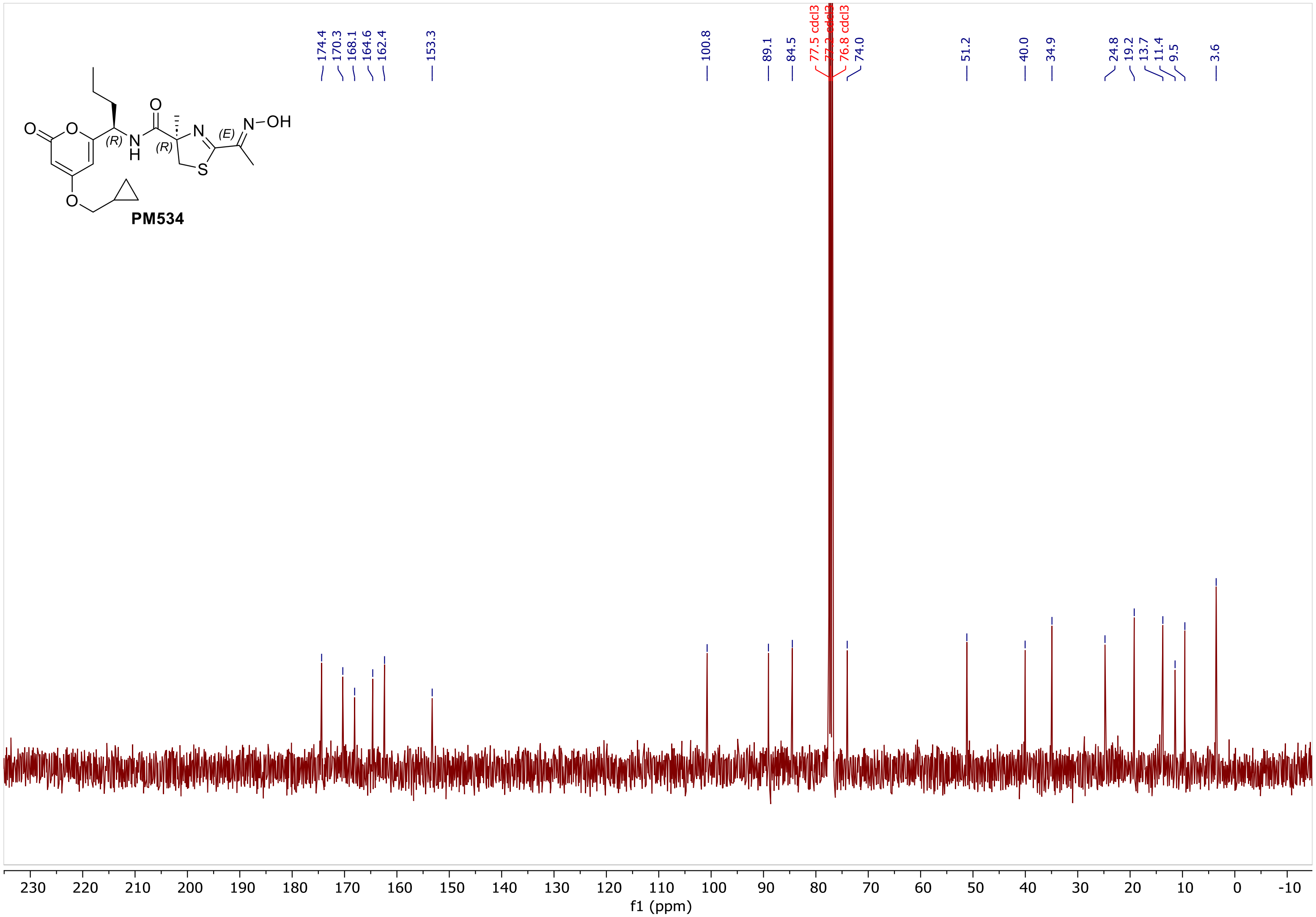

**Figure S31.** $^{1}$H NMR spectrum of (*R*)-*N*-((*R*)-1-(4-(Cyclopropylmethoxy)-2-oxo-2*H*-pyran-6-yl)butyl)-2-((*Z*)-1-(hydroxyimino)ethyl)-4-methyl-4,5-dihydrothiazole-4-carboxamide (*Z*-**PM534**). (400 MHz, $CD_3OD$).

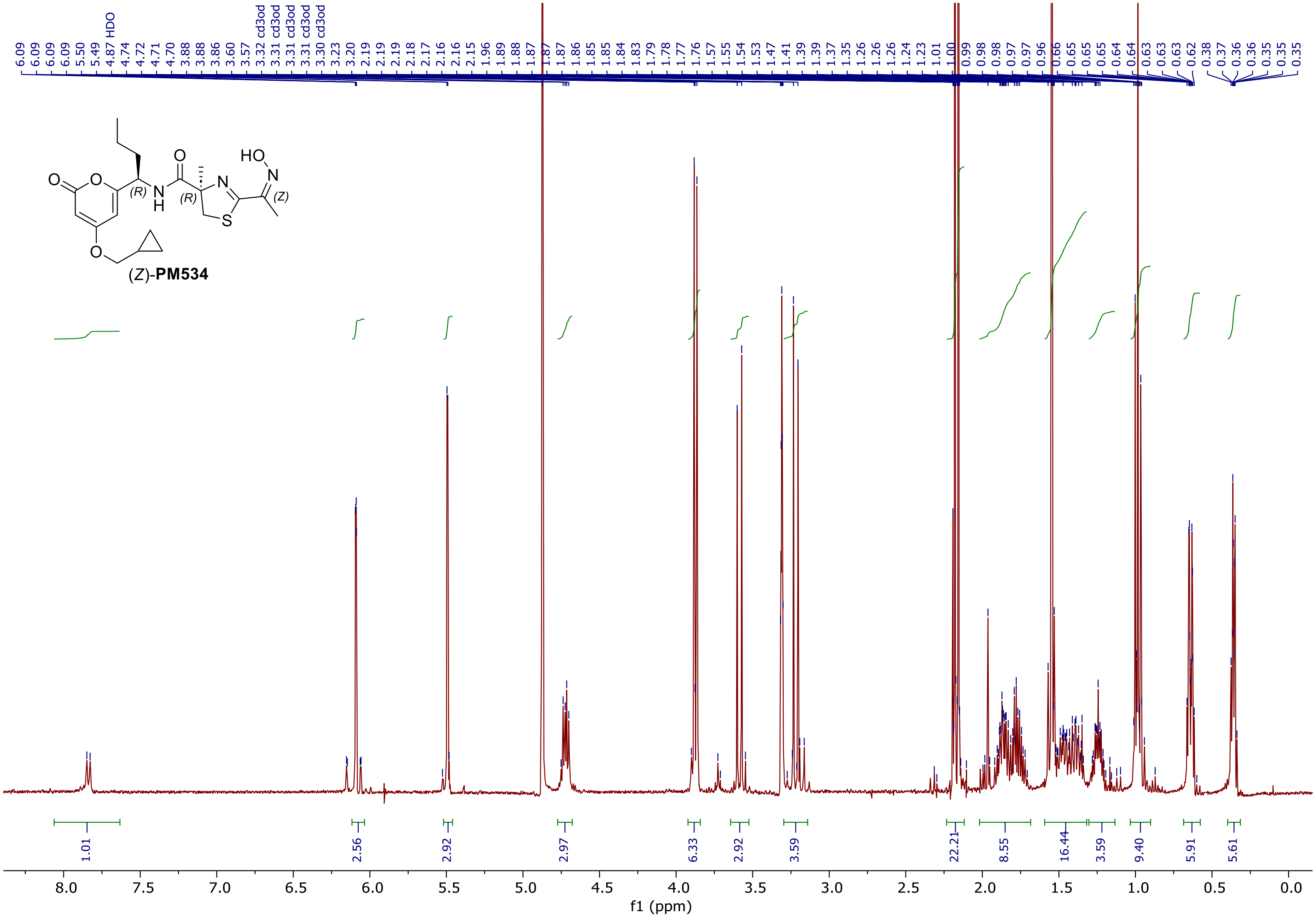

**Figure S32.** $^{13}$C NMR spectrum of (*R*)-*N*-((*R*)-1-(4-(Cyclopropylmethoxy)-2-oxo-2*H*-pyran-6-yl)butyl)-2-((*Z*)-1-(hydroxyimino)ethyl)-4-methyl-4,5-dihydrothiazole-4-carboxamide (*Z*-**PM534**). (100 MHz, $CDCl_3$).

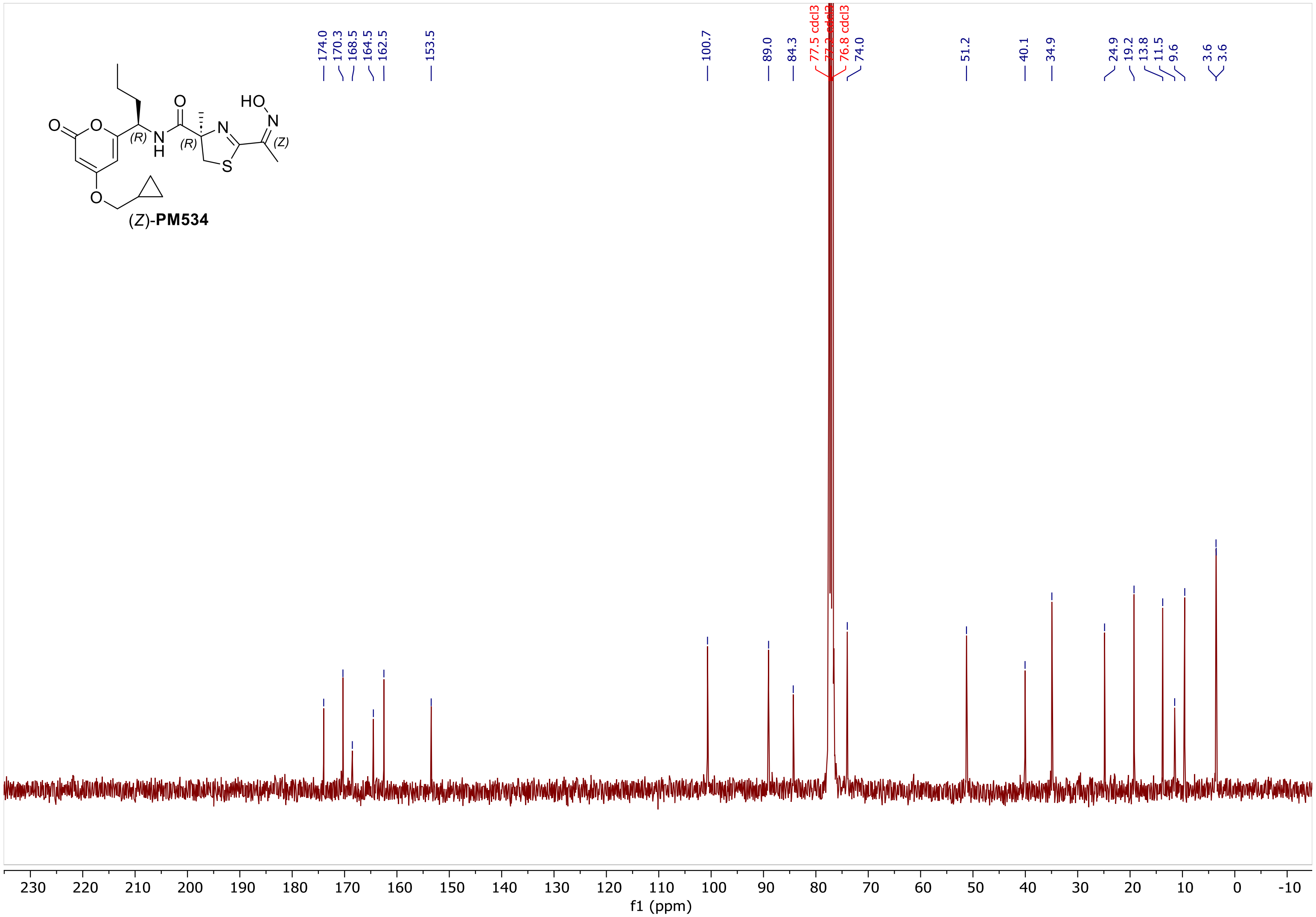

Supplement: Supplementary file 1 [file marinedrugs-24-00167-s001.zip › marinedrugs-4250276-supplementary.pdf]
